# Protocol to simulate crystalline Si-based single- and multi-junction solar cells under standard test and real-world conditions via MATLAB scripts

**DOI:** 10.1016/j.xpro.2024.103464

**Published:** 2024-12-03

**Authors:** Hesan Ziar

**Affiliations:** 1 Photovoltaic Materials and Devices (PVMD) Group, Electrical Sustainable Energy (ESE) Department, Electrical Engineering, Mathematics, and Computer Science (EEMCS) Faculty, Delft University of Technology, Mekelweg 4, 2628 CD Delft, the Netherlands

**Keywords:** Computer sciences, Energy, Environmental sciences, Physics

## Abstract

Silicon solar cells are the basis of the photovoltaic industry; thus, understanding their performance limits and parameter optimization under various working conditions is important. Here, we present a protocol for simulating mono-facial and bifacial silicon solar cells as well as 2-terminal double-junction X-on-silicon solar cells. We describe steps for resource gathering, input data preparation, running simulation scripts, and results visualizations. This protocol can be extended to simulate 3- and 4-terminal solar cells and more than two junctions.

For complete details on the use and execution of this protocol, please refer to H. Ziar.^1^

## Highlights

• Instructions for preparing data and MATLAB codes for photovoltaic cell simulation
• Guidance on simulating mono-facial, bifacial, and tandem silicon PV cells
• Steps for solar PV cell simulation under outdoor as well as standard test conditions
• Technique for visualizing optimum PV cell parameters for geographical locations

## Abstract (editor highlights)

Publisher’s note: Undertaking any experimental protocol requires adherence to local institutional guidelines for laboratory safety and ethics.

## Graphical abstract

**Figure:**
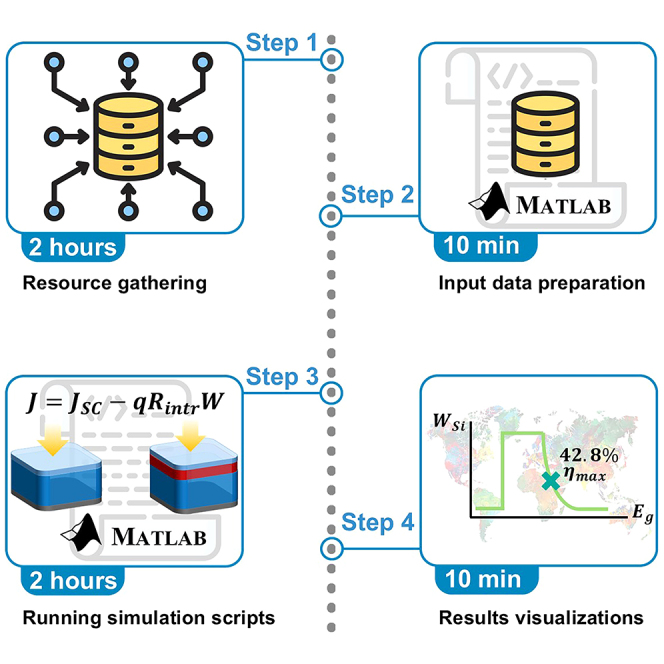

## Before you begin

The key part of solar systems for generating electricity is photovoltaic (PV) cells. PV cells convert photons of light to electrons of electricity. The main material used to produce solar cells is silicon. Since solar photovoltaic manufacturing is currently heavily based on crystalline silicon as the core material, simulating such cells is of great importance. For the same reason (the current industry heavily depends on crystalline silicon), it is not plausible to exclude double-junction silicon-based solar cells from future perspectives, when tandem cell architecture (solar cells with different materials on top of each other to use sunlight more efficiently) come into the picture. The sunlight spectrum and temperature that solar cells experience under outdoor environments are different from standard test conditions (STC). Therefore, it is crucial to simulate how they perform under outdoor conditions as well. This will help tailor the design and manufacturing of future solar photovoltaic cells for the geographical installation market and further down the road have more resource-efficient solar cells in the future.

### Scope

The primary aim of this protocol is to facilitate a simulation platform that researchers and manufacturers could use to adjust their silicon solar cell designs for target geographical locations. Besides, it could facilitate educators with a tool for teaching purposes and illustration of solar cell concepts topics. The protocol below describes the specific steps for simulating (i) single-junction silicon solar cell and (ii) 2-terminal double-junction cell with silicon as the bottom cell, under STC and worldwide outdoor conditions. With slight adjustment, we show how to use this protocol for bifacial silicon cells as well.

For the top cell, we neglect the non-radiative recombination and follow the Shockley-Queisser (SQ) approach. This is because the main candidate for the top cell in the industry is perovskite which is a direct band-gap material, contrary to crystalline silicon, where the radiative is the main recombination mechanism, which is considered in the SQ approach. For the bottom silicon cell, 1-dimensional semi-conductor physics equations are implemented and the narrow base assumption is used. Measured properties of crystalline silicon and free carrier absorption, incomplete ionization, photon recycling, and band-gap narrowing effects are considered.

### Hardware and software requirements

MATLAB and SMARTS software packages are used in this protocol. MATLAB is used to write the scripts for the implementation of the model while SMARTS, which stands for Simple Model of the Atmospheric Radiative Transfer of Sunshine, is used to generate the sunlight spectrum based on atmospheric conditions.^2^^,^^3^^,^^4^ MATLAB requires purchasing a license while SMARTS is free of charge but registration and accepting licensing terms are required before downloading the software.

The hardware must be capable of installing MATLAB and SMARTS software packages. Besides, it must have at least 8 TB of storage space, which is necessary while generating one year of hourly sunlight spectrum from 280 nm to 4000 nm for 15325 geographical coordinates (land locations with a resolution of 1° × 1°). Higher spatial and/or temporal resolution would demand more storage capacity.

The present protocol and the associated timings are based on the assumption of using a conventional office PC with the following system configurations: Intel Xeon CPU E5–3.5 GHz processor, 16 GB RAM, 64-bit operating system. More computational power would reduce the reported timings.

***Note:*** The protocol reports a minimum to a maximum needed time for the protocol part (in the graphical abstract only the minimum timings were mentioned though). The minimum timing is the time needed for only simulating under standard test conditions (STC) whereas the maximum timing indicates what is needed to simulate for all 15325 geographical locations on Earth.

***Note:*** SMARTS, which is widely in use in the solar and PV industry, only simulates irradiance under clear-sky conditions. To simulate the influence of clouds, the BRL model is used in this work.^5^ BRL is an irradiance decomposition model that estimates the share of the diffuse component in the total irradiance based on a few input parameters. The direct and diffuse ratios (fraction of the total sunlight that is direct or diffuse) found in the BRL model were applied to the direct and diffuse sunlight spectra generated by SMARTS. In this way, the diffuse part of the spectrum, which is influenced by the presence of clouds, is considered in this protocol.^6^

## Key resources table

The table below lists the ingredients needed to follow the protocol. When the aim is to simulate the solar cell only under STC, then only the ASTM G-173-03 is needed from the Deposited data list. The rest of the Deposited data items are for when performance under outdoor conditions is simulated.

**Table:** 

REAGENT or RESOURCE	SOURCE	IDENTIFIER
**Deposited data**		

ASTM G-173-03 tables	ASTM International	https://www.nrel.gov/grid/solar-resource/spectra-am1.5.html
Air temperature at the earth’s surface	NASA (GLDAS)	https://disc.gsfc.nasa.gov/datasets/GLDAS_NOAH10_3H_EP_2.1/summary
Relative humidity	NASA (GLDAS)	https://disc.gsfc.nasa.gov/datasets/GLDAS_NOAH10_3H_EP_2.1/summary
Surface pressure	NASA (GLDAS)	https://disc.gsfc.nasa.gov/datasets/GLDAS_NOAH10_3H_EP_2.1/summary
Precipitable water	NASA (CERES)	https://ceres-tool.larc.nasa.gov/ord-tool/jsp/SYN1degEd41Selection.jsp
Ozone total-column	NASA (CERES)	https://ceres-tool.larc.nasa.gov/ord-tool/jsp/SYN1degEd41Selection.jsp
Snow coverage	NASA (CERES)	https://ceres-tool.larc.nasa.gov/ord-tool/jsp/SYN1degEd41Selection.jsp
Aerosol optical thickness at 550 nm	NASA (CERES)	https://ceres-tool.larc.nasa.gov/ord-tool/jsp/SYN1degEd41Selection.jsp
Surface shortwave down flux	NASA (CERES)	https://ceres-tool.larc.nasa.gov/ord-tool/jsp/SYN1degEd41Selection.jsp
Top-of-atmosphere (TOA) shortwave flux	NASA (CERES)	https://ceres-tool.larc.nasa.gov/ord-tool/jsp/SYN1degEd41Selection.jsp
Altitude	JISAO	http://research.jisao.washington.edu/data_sets/elevation/
Urban extents grid	NASA (SEDAC)	https://sedac.ciesin.columbia.edu/data/set/grump-v1-urban-extents/
Albedo spectra	NASA (ECOSTRESS)^a^	https://speclib.jpl.nasa.gov/download
Population density	NASA (NEO)	https://neo.gsfc.nasa.gov/view.php?datasetId=SEDAC_POP

**Software and algorithms**		

SMARTS 2.9.5	Solar Consulting Services	https://www.nrel.gov/grid/solar-resource/smarts.html
MATLAB 2020b	MathWorks	https://nl.mathworks.com/products/matlab.html

**Other**		

Conventional CPU	Intel	Xeon CPU E5- 3.5 GHz
Storage	Western Digital	WD Elements Portable 2 × 4 TB

a Formerly known as ASTER spectral library.

## Step-by-step method details

### Part 1: Acquiring software packages and preparing datasets

**Timing: 2 h to 2 weeks**

In the first step, we install MATLAB and download SMARTS as well as the datasets listed in the key resources table. Further, we tailor them based on the simulation needs and preferences.

1. Download and install MATLAB following the instructions provided on this link: https://nl.mathworks.com/products/matlab.html.

***Note:*** You may install a preferred version of MATLAB. The author implemented the protocol on MATLAB versions 2019, 2020, 2021, and 2022, and they all worked.

**CRITICAL:** When installing MATLAB, make sure that the following toolboxes are ticked and installed: Curve fitting toolbox, Optimization toolbox, Global optimization toolbox, and Image Processing toolbox. The first toolbox is needed when we extrapolate the top and bottom cell's current density vs voltage curves to find the matching current density between the cells in a tandem structure. The second and third toolboxes are needed when we try to find the optimum parameters of the silicon-based tandem solar cell at all geographical locations. Using an optimization algorithm instead of calculating the tandem cell performance at all combinations of top and bottom cell parameters requires an unmanageable simulation time, especially when all geographical locations are studied. The fourth toolbox helps with visualization and interaction with the global maps that will be generated at the end of the protocol. If any of these toolboxes are not installed, then during the execution of MATLAB scripts and functions presented in this protocol there will be errors such as: 'optimoptions' requires Optimization Toolbox.

2. Download SMARTS 2.9.5: Simple Model of the Atmospheric Radiative Transfer of Sunshine via this link: https://www.nrel.gov/grid/solar-resource/smarts.html.
  ***Note:*** Users must register before downloading the SMARTS. Registration is free.
  a. The distribution package is compressed. After decompression, a SMARTS_295_PC folder is created.
  b. Keep a record of SMARTS_295_PC folder directory.
    ***Optional:*** A few rounds of trial and error with SMARTS software, filling out different virtual “Input Cards”, and checking the generated output .txt files help with a better identification and implementation of the protocol. Start with the Excel version of the SMARTS to get a better understanding of the “Input Cards”. Alternatively, have a quick look at the user manual which is in SMARTS_295_PC folder.
3. Go to https://www.nrel.gov/grid/solar-resource/spectra-am1.5.html,
  a. scroll down the page and click on the spreadsheet  link to download the Reference Air Mass 1.5 Spectra in .xls format.

***Note:*** The Excel file name is astm173 and contains four columns: wavelength (nm), extraterrestrial spectral irradiance (W/m-2/nm), global total spectral irradiance (W/m-2/nm), and direct normal spectral Irradiance (W/m-2/nm).

***Note:*** The wavelength step varies along the data. From 280 nm to 400 nm is every 0.5 nm, from 400 nm to 1700 nm is every 1 nm, then there is a step to 1702 nm and 1705 nm, and from 1705 nm to 4000 nm is every 10 nm.

**CRITICAL:** For 1° × 1° global mapping visualizations similar to those in H. Ziar 2024 in Joule,^1^ we cover latitude coordinates from −60 South to +90 North, removing the Antarctic peninsula. This will end up with 15325 land locations. To keep the consistency between the data files, some of them have to be tailored after downloading.

**CRITICAL:** All the steps below until Part 2 are skipped when the solar cell simulation is only for STC.

4. Go to https://disc.gsfc.nasa.gov/datasets/GLDAS_NOAH10_3H_EP_2.1/summary,
  a. Click on  Subset/get data.
  b. In the opened window click on the Download Method tab and tick this option: Get File Subsets using the GES DISC Subsetter.
  c. Then click on the Select Variables tab.
  d. In the opened drop-down list, select Psurf_f_inst = Surface air pressure (Pa), Tair_f_inst = Air temperature (K), and Qair_f_inst = Specific humidity (kg kg-1).
  e. The special resolution of the data is 1° × 1°, with 3-h intervals. Refine the Data Range and Region according to your time duration and geographical region of interest using the Refine Region tab.
  f. Then, click on the Get Data button to download the data.^2^

**CRITICAL:** Each downloaded file contains a single time interval, a bulk downloader or an extension for mass download is recommended. The files are .nc (NetCDF) and can be opened in MATLAB using ncinfo and ncread. Alternatively, one can use Panoply software tool for opening, converting, and visualizing .nc files. More information here: https://www.giss.nasa.gov/tools/panoply/download/

***Note:*** Since each value is the average of the past 3 h, when you select one year, the first file is from 01/January/year at 3:00 h and the last file is from 01/January/year+1 at 0:00 h.

**CRITICAL:** Mind the units! The temperature is given in Kelvin and SMARTS needs the input to be in °C, thus 273.15 must be subtracted from all temperature values. The pressure data is in Pascal and must be divided by 100 to convert it to mbar, which is the necessary unit for SMARTS. The humidity data is specific humidity, *q*, given in kg/kg. For SMARTS, the input needed is relative humidity, *RH*, and it is computed by the following equation.^7^^,^^8^^,^^9^^,^^10^*P* is pressure (in mbar), *T*_*0*_ is the reference temperature (273.15 K) and *T* is the temperature in Kelvin.

(Equation 1) $$RH\approx 0.263.\;q.\;p.\exp\;{\left(\frac{17.67\left(T-T_{0}\right)}{T-29.65}\right)}^{-1}$$

5. Go to https://ceres-tool.larc.nasa.gov/ord-tool/jsp/SYN1degEd41Selection.jsp.
  a. Under the Initial TOA and Surface Fluxes and Meteorological Parameters, select Initial Meteorological Parameters. Then select Precipitable Water, Column Ozone, and MATCH AOD @ 0.55 micron.
  b. Next, select Initial Surface Fluxes, then select Shortwave Down Flux, then untick all options except All Sky.
  c. Further, select Initial TOA Fluxes, then select Shortwave Flux and tick the All Sky.
  d. Under the Auxiliary Data, select Surface Data and then select Snow/Ice Percent Coverage.
  e. Under the Temporal Resolution, select the Hourly.
  f. Under the Spatial Resolution, select Regional (1° × 1° global grid) north 90°, West −180°, East 180°, South −60°
  g. Under the Satellite, select Terra+Aqua/NOAA20 Edition 4.1 (3/2000–3/2024).
  h. Under the Time Range, put dates in such a way that covers one year, for instance, from 01–2019 to 12–2019.
  i. Enter your email address.
    **CRITICAL:** For data files less than 2GB, immediate download is available. When the amount of data is more than 2GB, you need to make a free account, go to the shopping cart, and submit an order. The abovementioned CERES data specification and range (temporal hourly, 1° × 1° resolution) will be 11.34 GB. Figure 1 shows the screenshots of the steps in downloading CERES data.^11^

**Figure 1:**
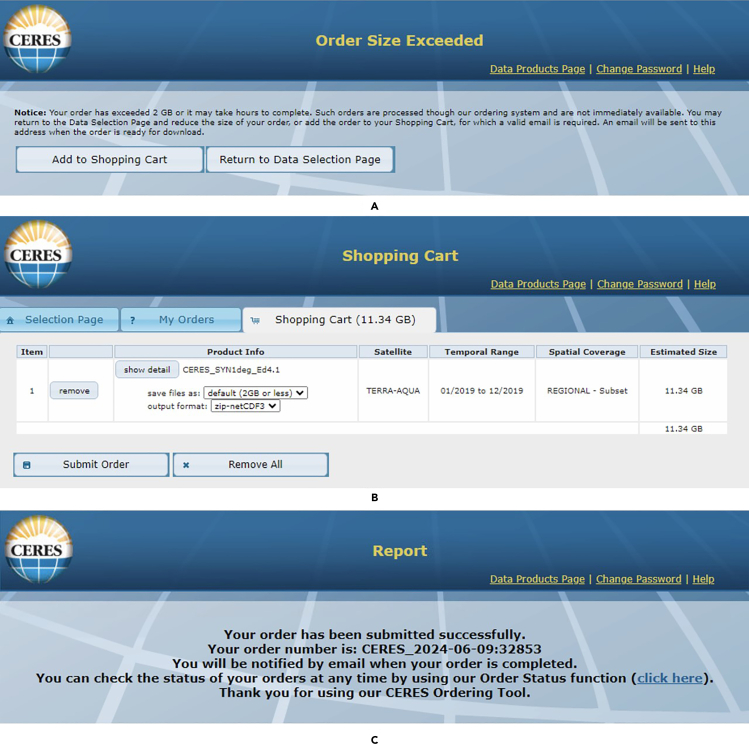
Screenshots of downloading CERES data
  j. Click on Submit Order. You will receive a notification saying that your order is being processed. You will receive an email containing download links in less than 0.5 h.
    **Pause point:** For 0.5 h.
    **CRITICAL:** The links will expire in 10 days. Mind a timely download.
  k. Download and unzip the data.
  l. Use ncinfo and ncread commands in MATLAB to read and convert the .nc data to .mat. See the code sample below.
    >> fileinfo = ncinfo("ExampleNCData.nc");  % take a look at variable
    % names and use appropriate variable name(s) in the next line
    >> variable=ncread("ExampleNCData.nc", "variable");
6. Go to http://research.jisao.washington.edu/data_sets/elevation/ and then click on 1-degree latitude-longitude resolution elevation (Rand Corporation / Scripps Institution of Oceanography). The file will be downloaded automatically (elev.1-deg.nc).
  a. Use ncinfo and ncread commands in MATLAB to read and convert the .nc data to .mat. See a sample code below.

>> fileinfo = ncinfo("elev.1-deg.nc");

>> Altit=ncread("elev.1-deg.nc", "data");

7. Go to https://sedac.ciesin.columbia.edu/data/set/grump-v1-urban-extents/, and click the Data Download tab.
  ***Note:*** You need to log in to download the data. A free account can be made.
  a. Select Region and Global on the Geography drop-down menus.
  b. Select .bil (other options: .ascii and gris) and 30″ on the Data Attribute drop-down menu.
  c. Select Urban Extents Grid on the Data Set drop-down menu.
  d. Click Download and a zipped folder containing the .bil data will be downloaded.
    **CRITICAL:** The downloaded matrix contains either of the following three integer values at each geographical location: 0 = Large water bodies, 1 = rural, and 2 = urban.
    **CRITICAL:** The downloaded file covers all longitude ranges but only covers latitude values from +84° North to −56° degrees South. Besides, it has a much higher resolution than 1° × 1°. Therefore, we do appropriate matrix adaptation and downsizing.
    **CRITICAL:** A .bil file can be opened in MATLAB using multibandread. The required input information for multibandread function can be found in the corresponding .hdr file that is downloaded together with the .bil data.
    ***Note:*** The following MATLAB code shows an example of reading and converting the file. We considered the data value (either 0, 1, or 2) at each integer geographical location while down-sizing the data matrix. Alternatively one could average over every 120 × 120 block of data, however, SMARTS needs an integer number for each geographical location to know if it is rural or urban. Figure 2 illustrates matrix adaptation and downsizing of the process of the global urban extents in the form of maps. This figure visualizes how changing the resolution, from high to low, influences the data.
    filename ='glurextents.bil';
    % From the .hdr file, following arguments for MUTLIBANDREAD function obtained.
    size = [16800 43200 1];   % [NROWS NCOLS NBANDS]
    precision = 'int8';     % from NBITS and PIXEL TYPE = int
    offset = 0;       % since the header is not included in this file
    interleave = 'bil';     % LAYOUT
    byteorder = 'ieee-le';    % BYTEORDER = I (refers to Intel order or little endian format)
    X_bil = multibandread(filename, size, precision, offset, interleave, byteorder);
    figure(1), imagesc(X_bil)   % Display the image file
    longitude=[60:120:43200];
    latitude=[60:120:16800];   % from N 83.5 to S -55.5 latitude degrees
    Urban_Extents(1:6,1:360)=0;  % 89.5 to 84.5
    for d=1:140
     for e=1:360
     Urban_Extents(d+6,e)=X_bil(latitude(d),longitude(e));
     end
    end
    Urban_Extents(147:150,1:360)=0;  % from N 89.5 to S 84.5 latitude degrees
    figure(2), imagesc(Urban_Extents)  % Display the image file

**Figure 2:**
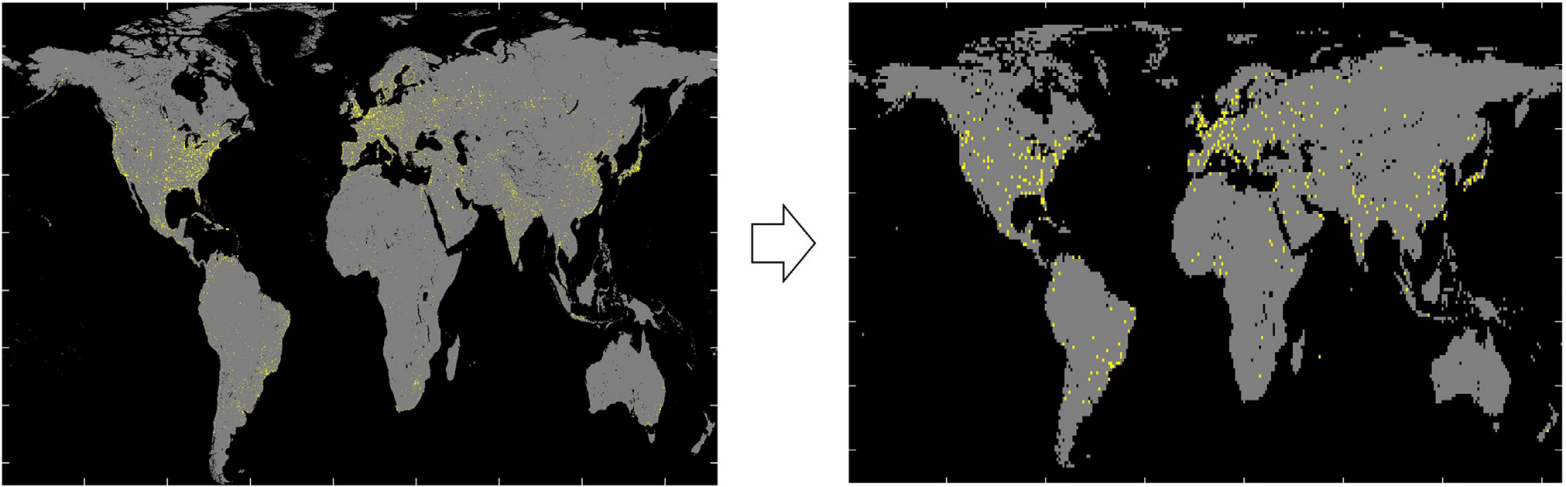
Visualization of the matrix adaptation and downsizing process of the global urban extents grid The figure on the left, obtained from the originally downloaded data, is converted through the process described in sub-steps 7.a to 7.d to obtain the data form that is compatible with the rest of the simulation framework, visualized by the right figure.
8. To acquire the albedo spectrum of the material placed underneath your PV modules, first go to https://speclib.jpl.nasa.gov/download.
  ***Note:*** In case you are simulating a scenario in which multiple materials are present under the PV module simultaneously, to model their overall effect, the view factor concept and algebra can be used (assuming that the reflection is Lambertian). For further readings and related examples, please see.^12^
  a. Select the material category that you would like to receive its spectral albedo. Multiple categories can be selected. Then click on the Checkout button.
  b. Fill in the checkout request form with your name, email address, institution, and country. Then click on the Submit button.
  c. In a few minutes, you will receive a confirmation email stating that your order will be processed within a week (or sooner). When the data link is received it remains valid for two weeks.
    **Pause point:** 1 week.
    **CRITICAL:** SMARTS uses the ECOSTRESS database. 66 material spectra are available in the SMARTS installation folder with a .dat format. They are in the subfolder ‘Albedo.’ If your material of interest is within the SMARTS list, instead of following sub-steps 8.a to 8.c, the SMARTS database can be used.
    **CRITICAL:** When using SMARTS to generate sunlight spectra on a target surface, Card 10 of SMARTS is for albedo input. By default, SMARTS uses ‘Light sandy soil’ as the material for albedo assessment, which is the same when standard atmospheric spectral data (astm173) is generated. Each selected material is known with a number in SMARTS that fills in the associated card. For Light sandy soil, it is 38, and for ‘Fresh dry snow’ it is 3. In the SMARTS installation folder, these two materials are named ‘LiteSoil’ and ‘FineSnow’.
    ***Note:*** In our global solar cell simulation, we use ‘Light sandy soil’ and ‘Fresh dry snow’ under the following conditions: when, at a time interval, more than 50% of the geographical location is covered with snow (from the data in step 5), we select ‘Fresh dry snow’ in SMARTS and otherwise, we select ‘Light sandy soil’. This means filling Card 10 of SMARTS with 3 and 38, respectively.
9. Go to https://neo.gsfc.nasa.gov/view.php?datasetId=SEDAC_POP and then click on ‘the SEDAC site’.
  a. From the Temporal, FileFormat, and Resolution tabs select your preferred options. Here we select single year (2020), ASCII, and 60 Min/1 Degree respectively.
  b. Click on Create Download.
  c. Click on the link provided below the Data Packages.
  d. Unzip the downloaded file, and open the .asc file, for instance with Notepad. You will see six lines of general information (ncols, nrows, xllcorner, yllcorner, cellsize, NODATA_value) and then the data. The no data (oceans and water areas) is identified with −9999.
  e. Import the data to Excel and MATLAB for further adjustment and use.
    **CRITICAL:** In the MATLAB codes provided in this protocol, whenever loading data is needed, it is commented as ***% in supplementary dataset*** in the code, indicating that the corresponding data can be found in the supplementary dataset. Please see the resource availability section to find the link to the supplementary dataset.
    load ('land_coord_index_population_density.mat');    % in supplementary dataset
    load ('land_coord_index.mat');     % in supplementary dataset
    k = 1;
    for i = 1:length(Land_coord_index)
     check=0;
     for j = 1:length(population_vector)
     if Land_coord_index(i,1) == population_density_matrix(j,1) && Land_coord_index(i,2) == population_density_matrix(j,2)
     Result (k,:) = population_density_matrix(j,:);
     k = k+1;
     check = check+1;
     end
     end
     if check == 0 % if there were no common geographical coordinate
          % in the two files, here it finds the closest
          % geo- graphical point to assign the population
          % density to that coordinates
     geo_difference (:,1) = abs(population_density_matrix(:,1)- Land_coord_index(i,1));
     geo_difference (:,2) = abs(population_density_matrix(:,2)- Land_coord_index(i,2));
     distance = sqrt((geo_difference(:,1).ˆ2) + (geo_difference(:,2).ˆ2));
     %[indx,indy]=find(distance==min(distance));
     [val, ind] = min(distance);
     Result (k,:) = population_density_matrix(ind,:);
     k = k+1;
     end
    end
    save 'Result'
    **CRITICAL:** The population density data covers 360 degrees of longitude and 180 of latitude. However, our study does not include the Antarctic peninsula which means we only cover latitude coordinates from −60 South to +90 North. This means we have 30 extra rows in the population density data (180–150). Therefore, we remove the last 30 rows of the downloaded population density data. After removing water locations (data values of −9999) and identifying the row and column indexes using the find command in MATLAB, there still will be 16988 data points, which is more than 15325 of our base mapping resolution. The above code downsizes the matrix to a resolution consistent with the rest of the global data. Since this downsizing causes the overall global population to change, and as a result biases the population density, after downsizing, all data points have to be multiplied by 7.758/6.2354 which is the ratio of population count (the year 2020) before and after downsizing.

### Part 2: Generating spatial, spectral, and temporal input data for solar cell simulations

**Timing: 10 min to 7 weeks**

In Part 2, we aim to create hourly spectral irradiance for all land locations. We implement SMARTS, which uses a text file to import its input variables and simulation preferences. This .txt file can be altered to create a preferred specific scenario. We use the gathered data in Part 2 to alter the input .txt file, execute SMARTS, and extract the output data into a .mat file.

10. Import the astm173 Excel file (obtained in step 3) into MATLAB and make a .mat file.

**Table 1:** Description of MATLAB codes’ role in Part 2

Code name	Role
SMARTS MATLAB Processing script	Receives target time period and geographical region and runs the other 6 functions
SMARTS Parser function	Loads atmospheric data, creates the input .txt files for SMARTS, runs SMARTS and saves the .txt output files.
SMARTS Importer function	Extracts data from the .out.txt files and creates the .mat files.
SMARTS Zenith Importer function	Extracts Sun zenith values from the .out.txt files. It is needed because SMARTS automatically skips calculations when it is nighttime (zenith > 90°) yet we need to know which inputs yielded which output.
BRL Model function	Calculates the diffuse and direct ratio of global horizontal irradiance (GHI) for all time stamps.
Night Filter function	Filters out nighttime data, based on the zenith values extracted in SMARTS Zenith importer function.
BRL Ratios function	Calculates the share of direct and diffuse components of irradiance on a target tilted plane and accordingly weights the direct and diffuse spectra calculated via SMARTS.

**CRITICAL:** All the steps below until Part 3 are skipped when the solar cell simulation is only for STC.

***Note:*** The rest of this protocol part is governed by seven MATLAB codes: one main script and six functions. Table 1 describes the role and necessity of each code.

***Note:*** SMARTS generates 3 files after it is run: smarts295.ext.txt contains the tabulated spectral irradiance; smarts295.out.txt contains information about inputs, output, and errors, and smarts295.inp.txt is a copy of the Input Card values.

11. Copy and paste the scripts and functions provided below in MATLAB environment.
  a. Copy and paste the **SMARTS MATLAB Processing script** in MATLAB environment.
    %% SMARTS MATLAB Processing script
    year=2019; month=(1:1:12); day=(1:1:31);
    hour=(0.5:1:23.5);   % 24:00 is 0:00 the next day; start at 0.5
    zone=0;      % time zone
    Total_time=(0.5:1:8760); % 8784 if it is a leap year
    days_2019=(1:1:365);
    co_min = 1; co_max = 15325; % start and end coordinate indexes of the
              % target region. Adapt accordingly.
    load('Land_coord.mat')    % in supplementary dataset
    project_path = 'C:\DirectoryOfTheProjectData\'; % This is adjusted per user!
    %% SMARTS Parser function
    cd(project_path)
    filename1='am15g_empty_test.txt'; % in supplementary dataset
    SMARTS_parser(filename1, year, month, day, hour, zone, co_min, co_max)
    clear day zone filename1
    %% SMARTS Importer function
    cd(project_path)
    [SMARTS_irr_cs, wvlngth, L, hours] = SMARTS_importer(Land_coord, project_path, days_2019, co_min, co_max);
    save('wvlngth.mat', 'wvlngth', '-v7.3')
    %% SMARTS Zenith Importer function
    cd(project_path)
    [all_zenith_SMARTS, filenames_zen] = zenith_importer(Land_coord, project_path, days_2019, co_min, co_max);
    %% BRL Model function
    [df, dr] = BRL_model(Land_coord, month, hour, co_min, co_max);
    %% Night Filter function
    [input_df, input_dr, SMARTS_zenith, Time_day] = Night_filter_function(Total_time, all_zenith_SMARTS, df, dr, project_path, co_min, co_max);
    %% BRL Ratios function
    [DNI_1h_2019_all, DHI_1h_2019_all] = BRL_ratios(SMARTS_zenith, input_df, input_dr, co_min, co_max);
    disp('done');
    **CRITICAL:** Before running the SMARTS parser function, make sure there are no .ext.txt, .out.txt, or .inp.txt in your main SMARTS installation directory.
    **CRITICAL:** Be mindful of the project path and the directory. Tailor them in the code based on the file directory on your own PC.
  b. Copy the **function SMARTS_parser** from Methods S1 in the supplemental information file and paste it into the MATLAB environment.
    **CRITICAL:** The input .txt file is constituted of a series of lines representing as many virtual “Input Cards”. In SMARTS, the total number of Input Cards is variable, depending on the complexity of the requested calculations. This means the number of lines in the input .txt file is variable. Therefore, when you change the Input Cards, make sure that the line numbers in the .txt file remain fixed.
    **CRITICAL:** The Input Cards numbered: 4, 6, 8, 10–12, 14, 17, 19–29 are the same for all coordinates. These are already pre-filled in the text file 'am15g_empty_test.txt' as the default text. This makes the SMARTS parser function faster. If you change any of these cards, make sure that you also change them in the default text file as well. The Methods S2 in the supplemental information file contains the commands needed to adjust the aforementioned Input Cards.
  c. Copy the **function SMARTS_importer** from Methods S3 in the supplemental information file and paste it into the MATLAB environment.
  d. Copy the **function zenith_importer** from Methods S4 in the supplemental information file and paste it into the MATLAB environment.
  e. Copy the **function BRL_model** from Methods S5 in the supplemental information file and paste it into the MATLAB environment.
  f. Copy the **function Night_filter** from Methods S6 in the supplemental Information file and paste it into the MATLAB environment.
  g. Copy the **function BRL_ratios** from Methods S7 in the supplemental Information file and paste it into the MATLAB environment.
  h. Now run the **SMARTS MATLAB Processing script** which calls the 6 functions above to obtain the desired irradiance spectra for one year, every hour, at every geographical location.
    **CRITICAL:** It is strongly recommended to use parallel computing, for instance by dividing the geographical coordinates into several batches and running each batch separately. Generation of all hourly irradiance spectra for one geographical location takes ∼4 min with a conventional office PC, summing up to ∼42 days (7 weeks) for the whole globe of 15325 locations. It is recommended to use parallel PCs for simulation time reduction and storage space management.
    ***Optional:*** Based on the generated data, a (weighted) average of the irradiance spectra and temperature over a desired period of time can be made. In the following part, we use yearly average irradiance and temperature for each geographical location in the MATLAB codes.
    **CRITICAL:** The scripts and functions in Part 2 assume that the sunlight-receiving surface has the same tilt as the latitude of the geographical location and is perfectly due south in the northern hemisphere and perfectly due north in the southern hemisphere. If other tilts and/or orientations are desired, then appropriate adjustments must be made in the **SMARTS_parser function** and **BRL_ratios function**.

### Part 3: Simulating single- and double-junction silicon-based solar cells

**Timing: 2 h to 49 weeks**

Having the spectral irradiance and temperature at hand, in Part 3, we run the MATLAB code for modeling single and double-junction silicon-based solar cells. First, we present the script for single junction silicon solar cells and then for tandem cells. Where possible, the code lines are supported by explanatory comments that are highlighted in gray.

12. Copy and paste the script provided below in the MATLAB environment.
  ***Note:*** It calculates the achievable efficiency of ***single-junction crystalline silicon solar cell*** at a range of geographical locations defined by co_min and co_max (minimum and maximum geographical coordinates) by calling 8 other functions. It uses the data generated in Part 2.
  **CRITICAL:** Be mindful of the project path and the directory. Tailor them in the code based on the file directory on your own PC.
  a. Copy and paste the **Single-junction crystalline silicon solar cell simulation script** into the MATLAB environment.
    %% Single-junction crystalline silicon solar cell simulation script
    c = 299792458;    % Speed of light in vacuum (m/s)
    k = 1.38064852e-23;    % Boltzmann constant (m2 kg/s2 K)
    q= 1.60217662e-19;    % Electron charge (Coulombs)
    h = 6.62607004e-34;    % Plack constant (m2kg/s or Joule.Sec)
    addpath('C:\DirectoryOfTheProjectData\All world results\global silicon PV efficiency');
    load ('G_POA_mean_BRL_world.mat'); load('Land_coord.mat'); load ('wvlngth.mat');
    load ('Tair_C_Daily_avg_2019_input.mat');    % in supplementary dataset
    lamda_nm = wvlngth; lamda =1e-9∗lamda_nm; E = (h∗c)./lamda;
    N_donor = logspace(12,15,5);    % n-type doping concentration range
    N_acceptor = 0;    % p-type doping concentration range
    Width = [1:1:500]∗1e-6;    % Si thickness range searched for the optimum (in m)
    NOCT = 48;       % nominal operational cell temperature, adapt if needed
    Eg=1.12;        % Silicon bandgap
    Vstart=0.000; Vstep=0.001; Vend = Eg;
    V = [Vstart:Vstep:Vend];
    Result = zeros (length(Land_coord), 13);   % prealocation to save parameters
    co_min = 1; co_max = 15325;      % range of geographical locations
    bifacial = 1; % 1 for monofacial and 2 for bifacial simulations
    for LandCoordIndex=co_min:co_max
    E_global_lamda = G_POA_1h_2019_avg_raw_BRL_world{LandCoordIndex,1}; % irradiance
    T = 273.15 + Tair_C_Daily_avg_2019_input(LandCoordIndex,1) +...
    (((NOCT-20)/800))∗G_POA_mean_BRL_world(LandCoordIndex,1); % temp. Kelvin
    ni_0 = 5.29e19 ∗ ((T/300)ˆ2.54) ∗ exp(-6726/T) ;   % Temperature dependent intrinsic carrier concentration from K. Misiakos and Tsamakis, D., 1993.
    phi_lamda = ((q.∗lamda)./(h∗c)).∗(E_global_lamda); % converts spectral irradiance energy into spectral photon flux (C/m2 s)
    total_incoming_radiation = trapz(lamda_nm, E_global_lamda); % incoming radiation
    %% Pre-allocation
    Pmpp = zeros (length(N_donor), length(Width));
    eta = zeros (length(N_donor), length(Width));
    Jsc_mApercm2 = zeros (length(N_donor), length(Width));
    Voc = zeros (length(N_donor), length(Width));
    FF = zeros (length(N_donor), length(Width));
    delta_n_mpp = zeros (length(N_donor), length(Width));
    Tau_mpp = zeros (length(N_donor), length(Width));
    delta_n_oc = zeros (length(N_donor), length(Width));
    Vmpp = zeros (length(N_donor), length(Width));
    Photon_Rec_Width = zeros (length(Width), length(V));
    B_integral_Width = zeros (length(Width), length(V));
    BGN_values = zeros (length(N_donor), length(V));
    ni_eff_values = zeros (length(N_donor), length(V));
    delta_n_values = zeros (length(N_donor), length(V));
    minimum = zeros (1, length(Width));
    x = zeros (1, length(Width)); y = zeros (1, length(Width));
    %% loop
    for j = 1:1:length(N_donor)
    Nd = N_donor(1,j); Na = N_acceptor;
    [Nd_plus, Na_minus] = Incompelte_Ionization (Nd, Na, k, T, n, p);
    [n0, p0, n, p, delta_n, ni_eff, delta_Eg, Nd_plus(j,1), Na_minus(j,1), iid, iia] = Carrier_Statistics (Nd, Na, T, V, ni_0, Eg);
    BGN_values (j,:) = delta_Eg; ni_eff_values (j,:) = ni_eff;
    delta_n_values (j,:) = delta_n; iid_values (j,:) = iid; iia_values (j,:) = iia;
    [mu_e, mu_h, sigma_e, sigma_h, sigma, rho] = Carrier_Mobility (Nd, Na, n, p, T);
    [alpha_bb, n_r] = Silicon_Optical_Constants (T);
    [alpha_FCA_n,alpha_FCA_p] = Free_Carrier_Absorption (Nd, Na, T, V, n, p);
    alpha_FCA = alpha_FCA_p + alpha_FCA_n;
    for i = 1:1:length(Width)
    W = Width(1,i);
    [R_intr, Photon_Rec, B_integral] = Intrinsic_Recombination_Rate (Nd, Na, T, V, W, n, p, n0, p0, delta_n, ni_eff);
    Photon_Rec_Width(i,:) = Photon_Rec; B_integral_Width(i,:) = B_integral;
    A_bb = (alpha_bb∗ones(1,length(V))) ./ (alpha_bb∗ones(1,length(V)) + alpha_FCA + (bifacial./(4.∗((n_r.ˆ2)∗W∗1e+2)))∗ones(1,length(W))); % from T. Tiedje, et al, IEEE Trans. Electron Device, 1984.
    J_L_mApercm2 = (1e3/1e4).∗trapz(lamda_nm, A_bb.∗(phi_lamda∗ones(1,length(V)))); % 0.1 coefficient is to convert A/m2 to mA/cm2
    J_mApercm2 = (J_L_mApercm2) - 1000∗q∗((W∗1e+2).∗real(R_intr));    % and that leads to yield complex numbers for R sometimes, therefore I coded it as real(R) here! % qWR is in A/cm2, multiplied by 1000 to convert to mA/cm2, & from A. Richter et al., IEEE JPV (2013).
    J_Aperm2 = J_mApercm2∗10; P = J_Aperm2.∗V; Pmpp(j,i) = max(P);    % Pmpp in W/m2
    eta(j,i) = (Pmpp(j,i))./total_incoming_radiation;
    Jsc_mApercm2 (j,i) = max (J_mApercm2); minimum (1,i) = min(abs(J_mApercm2));
    x(1,i)=find(abs(J_mApercm2) == minimum(1,i));
    Voc (j,i) = Vstart+(Vstep.∗(x(1,i)-1));
    FF (j,i) = Pmpp(j,i) / (10.∗(Voc(j,i)∗Jsc_mApercm2(j,i)));
    y(1,i)=find(P == Pmpp(j,i)); delta_n_mpp(j,i) = delta_n(1, y(1,i));
    Tau_mpp(j,i) = delta_n_mpp(j,i)/R_intr(1, y(1,i));
    delta_n_oc(j,i) = delta_n(1, x(1,i));
    Tau_oc(j,i) = delta_n_oc(j,i)/R_intr(1, x(1,i)); Vmpp(j,i) = V(1, y(1,i));
    [LandCoordIndex,j,i]    % just to see at what running stage the code is
    Resistivity_sc (j,i)= rho(1,1);     % Resistivity at V=0 (ohm.cm)
    Resistivity_oc (j,i)= rho(1,x(1,i));    % Resistivity at V=VOC (ohm.cm)
    Resistivity_mpp (j,i)= rho(1,y(1,i));    % Resistivity at V=VMPP (ohm.cm)
    L_mpp(j,i) = sqrt((k∗T/q)∗(mu_h∗Tau_mpp(j,i)));     % Minority carrier diffusion length at Vmpp (cm)
    L_oc(j,i) = sqrt((k∗T/q)∗(mu_h∗Tau_oc(j,i)));      % Minority carrier diffusion length at Voc (cm)
    end
    end
    %% Saving target variables
    [index_row,index_column]=find(eta==max(max(eta)));
    Result(LandCoordIndex,:) = [eta(index_row,index_column) Voc(index_row,index_column) Jsc_mApercm2(index_row,index_column) 100∗FF(index_row,index_column)...
     Vmpp(index_row,index_column) delta_n_mpp(index_row,index_column) delta_n_oc(index_row,index_column) 1e6∗Width(1,index_column)...
     BGN_values(index_row,index_column) ni_eff_values(index_row,1) Nd_plus(index_row,1)-Na_minus(index_row,1) Resistivity_mpp(index_row,index_column)...
     (1e4∗L_mpp(index_row,index_column))/(1e6∗Width(1,index_column))];
    End
    %% Wrapping up
    clearvars -except Result co_min co_max
    save('C:\DirectoryOfTheProjectData\All world results\global silicon PV efficiency\Result_siliconPV_global_efficiency.mat');
    mkdir(fullfile(sprintf('%s_%d-%d', 'Results_1x1',co_min,co_max))); % makes a folder including range of the target coordinates
    movefile ('Result_siliconPV_global_efficiency.mat', fullfile(sprintf('%s_%d-%d', 'Results_1x1',co_min,co_max))) % moves all .mat files to the result folder
    ***Note:*** By removing the main for loop of the code and co_min and co_max variables and their associated lines, one can use the Single-junction crystalline silicon solar cell simulation script code for only STC as well. Minor adjustments might be necessary.
    ***Note:*** The above script and associated functions are written for an n-type silicon solar cell. However, they can be conveniently adapted to a p-typed cell as well by adjusting the relevant parameters.
    **CRITICAL:** This script can be used for bifacial simulations as well by changing the parameter bifacial from 1 to 2 in the code. Mind the calculated irradiance on the module for bifacial simulation as the input irradiance to the code should be the sum of the front and the rear irradiances.
    **CRITICAL:** Keep the voltage step resolutions high (very small steps) otherwise there will be spikes in the graphs when plotting the solar cell parameters, either in single or double junction cells. The higher the voltage step resolution the smoother the results graphs but the higher the simulation time, thus tailor it according to your simulation preferences.
    ***Note:*** When you have limited time and only aim at the maximum efficiency, only run at the lowest dopant density level as the maximum efficiency happens at the border of undoped silicon.
    ***Optional:*** The present framework is based on several key research works. It is recommended to go through the publications listed in Figure 3, which shows the timeline of the most important papers that were used to generate the codes provided here.^13^^,^^14^^,^^15^^,^^16^^,^^17^^,^^18^^,^^19^^,^^20^^,^^21^^,^^22^^,^^23^^,^^24^^,^^25^^,^^26^^,^^27^^,^^28^ It is strongly recommended to read the EXPERIMENTAL PROCEDURES section of H. Ziar,^1^ where the technical flow of the simulation and the most important equations are discussed. This will facilitate probable troubleshooting and partial cross-validation within the steps of the present protocol.
  b. Copy the **Incompelte_Ionization function** from Methods S8 in the supplemental information file and paste it into the MATLAB environment.
    ***Optional:*** Incomplete ionization increases by dopant density and since the maximum efficiency of crystalline silicon cell happens at the border of undoped silicon, the incomplete ionization function can be skipped for higher simulation speed.
  c. Copy the **Carrier_Statistics function** from Methods S9 in the supplemental information file and paste it into the MATLAB environment.
  d. Copy the **Carrier_Mobility function** from Methods S10 in the supplemental information file and paste it into the MATLAB environment.
  e. Copy the **Silicon_Optical_Constants function** from Methods S11 in the supplemental information file and paste it into the MATLAB environment.
  f. Copy the **Free_Carrier_Absorption function** from Methods S12 in the supplemental information file and paste it into the MATLAB environment.
  g. Copy the **Intrinsic_Recombination_Rate function** from Methods S13 in the supplemental information file and paste it into the MATLAB environment.
  h. Copy the **Photon_Recycling function** from Methods S14 in the supplemental information file and paste it into the MATLAB environment.
  i. Copy the **Band_Gap_Narrowing function** from Methods S15 in the supplemental information file and paste it into the MATLAB environment.
  j. Run the **Single-junction crystalline silicon solar cell simulation script** in MATLAB environment.
    **CRITICAL:** Simulating single-junction crystalline silicon solar using the above MATLAB codes takes ∼6 min for each geographical location. This will add up to 9 weeks for all 15325 locations using one regular office PC. It is, therefore, strongly recommended to use high-power PCs or parallel computing. Another solution to reduce the computation time is to run the code only at a very low doping concentration (N_dop_ = 10^12^) as in almost all cases that is where the maximum efficiency happens. This will reduce the simulation time for each geographical location to 1 min, adding up to 1.5 weeks for the whole globe.

**Figure 3:**
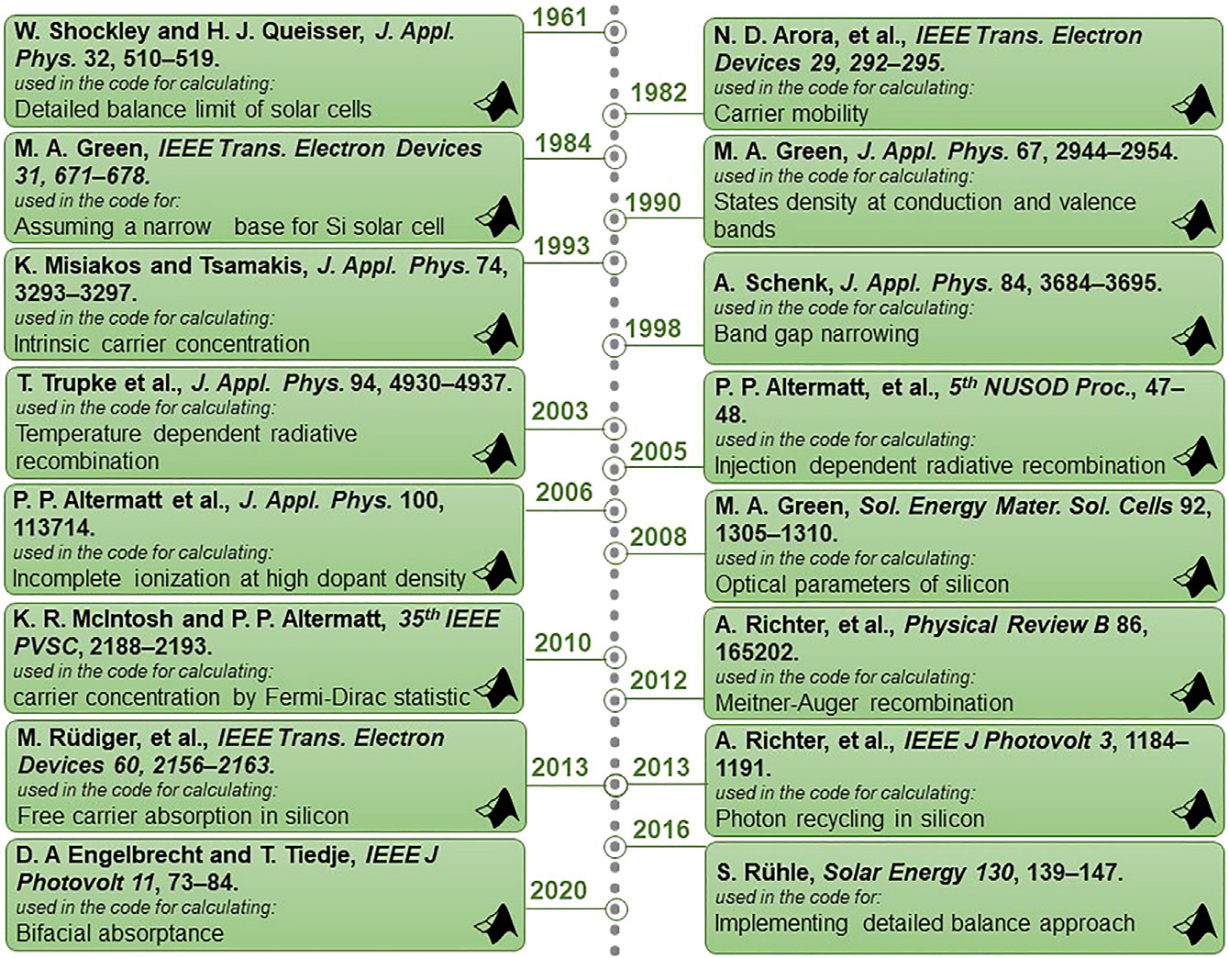
Timeline of the key influential scientific publications on silicon-based solar cell simulations implemented in this protocol within the MATLAB scripts
13. Copy and paste the scripts provided below into the MATLAB environment.
  ***Note:*** It calculates the achievable efficiency of ***double-junction crystalline silicon-based solar cell*** at a range of geographical locations defined by co_min and co_max (minimum and maximum geographical coordinates) by calling 8 other functions. It uses the data generated in Part 2.
  ***Note:*** For the top cell we consider the band gap as the variable parameter (which can be tuned for perovskite) and for the bottom cell we consider silicon wafer thickness (see Figure 4). That enables implementing the detailed balance approach for the top cell while preserving the silicon solar cell code presented in step 12 for the bottom cell. However, finding the optimum tandem cell parameters requires scanning a variable space that includes all possible combinations of the top and bottom cell parameters. This demands an extremely long simulation time for the whole globe. Therefore, to find the optimum tandem solar cell for each geographical location, we use an optimization algorithm to find the best combination of top cell band gap and silicon bottom cell thickness. Particle Swarm Optimization (PSO) is selected due to its easy implementation in MATLAB and overall effectivity. The result coming from the PSO-based simulation of tandem solar cells has been compared with exact time-extensive simulations and an almost perfect match was found.
  **CRITICAL:** Be mindful of the project path and the directory. Tailor them in the code based on the file directory on your own PC.
  a. Copy and paste the **Particle Swarm Optimization for tandem solar cell script** in MATLAB environment.
    %% Particle Swarm Optimization for tandem solar cell script
    load ('bandgap_PSO.mat');      % in supplementary dataset
    co_min = 1; co_max = 15325;
    Result_PSO = zeros(co_max-co_min+1,7);       % prealocation
    OutputMessage_PSO = cell(co_max-co_min+1,1);     % prealocation
    for LandCoordIndex=co_min:1:co_max
    tic;
    lower_boundry = [bangap_PSO(LandCoordIndex,1)-0.05, 1, 1e12, LandCoordIndex];
    upper_boundry = [bangap_PSO(LandCoordIndex,1)+0.05, 1000, 1e12, LandCoordIndex];
    rng default % For reproducibility
    options = optimoptions('particleswarm', 'SwarmSize',20, 'MaxIterations',20, 'MaxStallTime', 10∗60, 'MaxStallIterations', 10, 'InertiaRange', [0.05,1.1], 'HybridFcn', @fmincon, 'FunctionTolerance',1e-6);
    [x,fval,exitflag,output] = particleswarm(@ObjFunc_PSO,4,lower_boundry,upper_boundry,options);
    elapsedTime = toc;
    Result_PSO(LandCoordIndex,:)=[round(elapsedTime/60) output.funccount 1-fval x];
    OutputMessage_PSO{LandCoordIndex,:} = output;
    end
    clearvars -except Result_PSO OutputMessage_PSO co_min co_max
    save('C:\ DirectoryOfTheProjectData\All world results\Tandem\Result_tandem_2T_PSO3_OptimumPoints.mat');
    mkdir(fullfile(sprintf('%s_%d-%d', 'Results_1x1',co_min,co_max))); % makes a folder including range of the target coordinates
    movefile ('Result_tandem_2T_PSO3_OptimumPoints.mat', fullfile(sprintf('%s_%d-%d', 'Results_1x1',co_min,co_max))) % moves all .mat files to the result folder
  b. Copy and paste the **ObjFunc_PSO function** into the MATLAB environment.
    function [ ObjFunc ] = ObjFunc_PSO(input_parameter)
    bandgap = round(input_parameter(1,1),2);
    Width = round(input_parameter(1,2))∗1e-6;
    N_donor = round(input_parameter(1,3));
    LandCoordIndex = round(input_parameter(1,4))
    load ('WorldData_OneTime_Load.mat');    % in supplementary dataset
    eta_tandem = tandem_2T_world_PSO(bandgap,Width,N_donor,LandCoordIndex,...
    lamda_nm,Tair_C_Daily_avg_2019_input,G_POA_mean_BRL_world,Coef_WeightAveToAve_GPoA,Coef_WeightAveToAve_Tair,G_POA_1h_2019_avg_raw_BRL_world)
    ObjFunc=1-eta_tandem;
    end
  c. Copy the **tandem_2T_world_PSO function** from Methods S16 in the supplemental information file and paste it into the MATLAB environment.
    ***Note:*** For the top cell a step function absorptance is assumed, to comply with the general SQ approach. However, one can define or import a different absorptance function into the **tandem_2T_world_PSO function**, especially when the top cell material and its absorptance function are known.
    ***Note:*** The comments shared between the **tandem_2T_world_PSO function** and **Single-junction crystalline silicon solar cell simulation script** are omitted to avoid duplications.
    **CRITICAL:** The resulting accuracy of tandem cell simulation is more susceptible to voltage steps than single junction silicon cell simulations. It is strongly recommended to keep the voltage step resolutions high (very small steps). We suggest a 0.2 meV voltage step for tandem solar cells.
  d. Run the **Particle Swarm Optimization for tandem solar cell script** in MATLAB environment.
    **CRITICAL:** 2-Terminal double-junction tandem solar cell calculations for each geographical location take ∼26 min. This will add up to almost 40 weeks with one regular office PC. It is, therefore, strongly recommended to use high-power PCs or parallel computing, for instance by dividing the geographical coordinates into several batches and running each batch separately.

**Figure 4:**
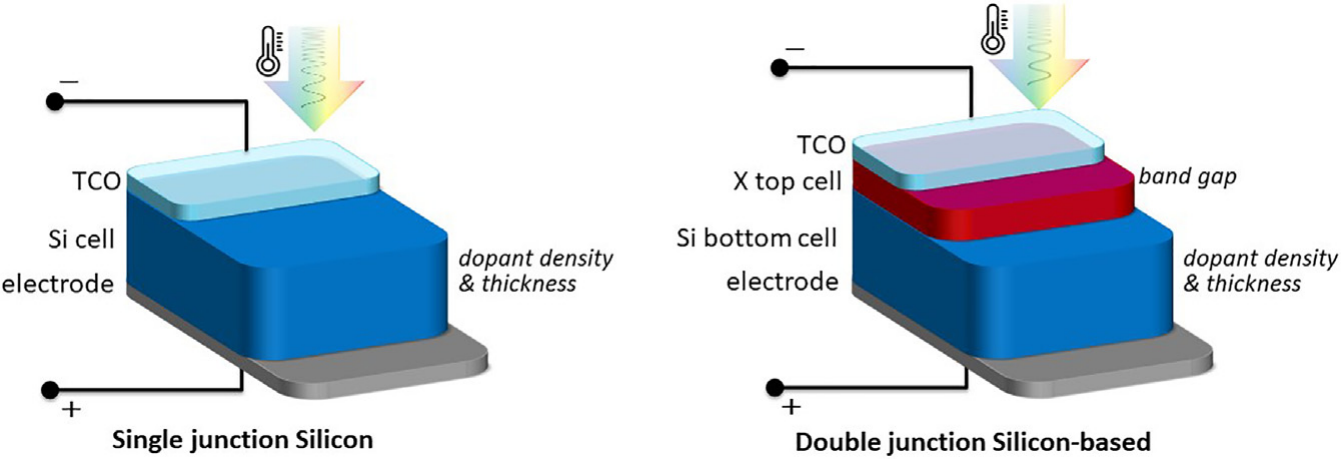
Architecture of single-junction silicon and double-junction two-terminal tandem solar cells simulated in the MATLAB code

### Part 4: Visualizing results as global maps

**Timing: 10 min to 1 h**

In this step, the numeric results obtained in the previous section are visualized in the form of global maps using a simple MATLAB script. MATLAB has specific commands for global mappings, such as the worldmap command, however, here, other syntaxes are used to make it transferable to other coding software packages.

14. Use the **Map maker** script below.

***Note:*** This will plot a colored global map of a parameter labeled Result that contains the numerical values of a desired parameter of the solar cell (such as efficiency, optimum wafer thickness, etc.) for all coordinates from 1 to 15325. By replacing the Result parameter in the above script one can plot it for other parameters of interest as well. Mind the graph title and axes labels by adjusting the title, xlabel, and ylabel parameters. This script adds a color bar and a distribution plot on the lower left side of the map as well. The scale of the distribution curve can be adjusted by tuning the fixed numbers in the plot command. When the solar cell simulation is done only for STC, the **Map maker** script is skipped and the user can instead plot the desired parameters of interest.

%% Map maker

map=zeros(180,360); Result_map=zeros(180,360);

for i=1:1:15325

map(Land_coord_index(i,1),Land_coord_index(i,2))=1;

Result_map(Land_coord_index(i,1),Land_coord_index(i,2))= Result(i,3);

end

Result_map = circshift(Result_map,[0 -10]);   % this shifts the whole map to the west by 10 longitude degrees. This is done to have the last piece of land in Russia attached to its main land (not to have in on the other side of the map)

%% plotting

Result_map(Result_map==0) = NaN;   % helps to get rid of the ocean points during plotting

f = figure(1);

f.Position = [1000 500 520 320];   % figure location and size (x0,y0,x,y)

set(gcf,'color','w');

contourf(Result_map, 'edgecolor','none',...

 'LevelList',min(Result(:,3)):0.0001:max(Result(:,3)));

colormap jet   % color distribution options(hot, parula, jet, gray etc.)

brighten(.1); ylim([20 180]); title('\eta (-)','fontSize', 12);

xlabel('Longitude ({\circ})','fontSize', 12);

ylabel('Latitude ({\circ})','fontSize', 12); set(gca,'fontSize',10)

DummyFigure = figure(2);  % this part opens a dummy figure, gets the countour info of the borders, and overlays it on the countour plot

[c,∼] = contour(isnan(Result_map),1,'-k','LineWidth',0.75);

c(c<1)=NaN; close(DummyFigure); figure(1); hold on; plot(c(1,:),c(2,:),'k');

h = gca; h.XAxis.Visible = 'off';h.YAxis.Visible = 'off'; % hiding x and y axes

ax1 = gca;        % tailoring the dimensions of the color bar

hcb=colorbar('SouthOutside');   % horizontal color bar on the figure’s lower side

ax1Pos = ax1.Position;

pos = hcb.Position; % gets the position of the color bar [left, bottom, width, height]. The left and bottom elements specify the distance from the lower-left corner of the figure or to the lower-left corner of the color bar.

pos(4) = 0.5∗pos(4);    % makes the color bar thinner by 50%

pos(3) = (0.25)∗pos(3); hcb.Position = pos; % applies the position

hcb.Limits = [min(Result(:,3)),max(Result(:,3))];   % specifying the min and max on the colorbar

[N,edges] = histcounts(Result(:,3),'binwidth',0.001);    % the last number defines the length of each bin

edges = edges(2:end) - (edges(2)-edges(1))/2; z = 1:(90-1)/(length(edges)-1):90;

plot(z, 38 + N/40, 'color', 'k','LineWidth',0.5); % The first number defines the up shift of the curve and the second number defines how high the distribution can go. Obtain it via trial and error for each map

print(gcf,'Result_jet_NoAxes.png','-dpng','-r900');    % printing

## Expected outcomes

The procedure described in the above 4 protocol parts yields multiple outcomes. These outcomes can be a solar cell parameter value under a specific irradiance and temperature condition (such as conversion efficiency at standard test conditions), or under a range of irradiance and temperature conditions (such as optimum design parameters of the cell for certain regions of the world). Depending on the research needs and goals, a researcher can tailor the protocol to his/her own research objectives. Moreover, the protocol published in this work can be used by academic educators to illustrate the basics of solar cells, concepts related to theoretical efficiency, basic designs of solar cells, geographical and weather dependency of solar cells performance, the contribution of different parts of irradiance spectrum on solar cells performance, etc. Here, a few expected result examples of the protocol are discussed.

The first example outcome is the Shockley-Queisser efficiency limit (and the associated parameters) versus the material band gap. In the **tandem_2T_world_PSO function**, the code section that simulates the top cell will give *exactly* the tabulated values presented in Table 1 of the work of S. Rühle^28^ under STC conditions. If one gets different values, troubleshooting is needed. A separate function for the detailed balance approach (Shockley-Queisser) is provided in Methods S17 of the supplemental information file for the reader’s convenience and quick use (**Detailed_Limit function**). Under STC, the maximum efficiency will be 33.15% and happens at 1.34 eV.

The second example outcome is the single-junction crystalline silicon solar cell’s maximum efficiency and the associated cell parameters under STC conditions for mono-facial and bifacial cells. For that, the **Single-junction crystalline silicon solar cell simulation script** is used at STC. Table 2 shows the expected numbers for efficiency (***η***), open circuit voltage (***V***_***OC***_), short circuit current density (***J***_***SC***_), fill factor (***FF***), voltage at maximum power point (***V***_***MPP***_), excess carrier concentration at maximum power point (***Δn***_***MPP***_) and open circuit (***Δn***_***OC***_), and the silicon wafer thickness (***W***).

**Table 2:** Expected output of important parameters for the single junction silicon solar cell (mono-facial and bifacial) under standard test conditions

	*η* (%)	*V*_*OC*_ (mV)	*J*_*SC*_ (mA/cm^2^)	*FF* (%)	*V*_*MPP*_ (mV)	*Δn*_*MPP*_ (cm^–3^)	*Δn*_*OC*_ (cm^–3^)	*W* (μm)
STC mono-facial	29.65	768.1	43.38	89.00	701.8	7.07×10^15^	2.57×10^16^	105
STC bifacial	29.14	756	43.37	88.89	690	5.62×10^15^	2.03×10^16^	207

**Table 3:** Code developers’ history

Code name	First version	Second version	Current version
SMARTS MATLAB Processing script	Tanja Post	-	Hesan Ziar
SMARTS Parser function	Cas de Mooij	Tanja Post	Hesan Ziar
SMARTS Importer function	Cas de Mooij	Tanja Post	Hesan Ziar
SMARTS Zenith Importer function	Tanja Post	-	Hesan Ziar
BRL Model function	Sandeep Mishra	Tim Stark	Hesan Ziar
Night Filter function	Tanja Post	-	Hesan Ziar
BRL Ratios function	Tanja Post	-	Hesan Ziar
All solar cell simulation scripts and functions	-	-	Hesan Ziar

**Figure 5:**
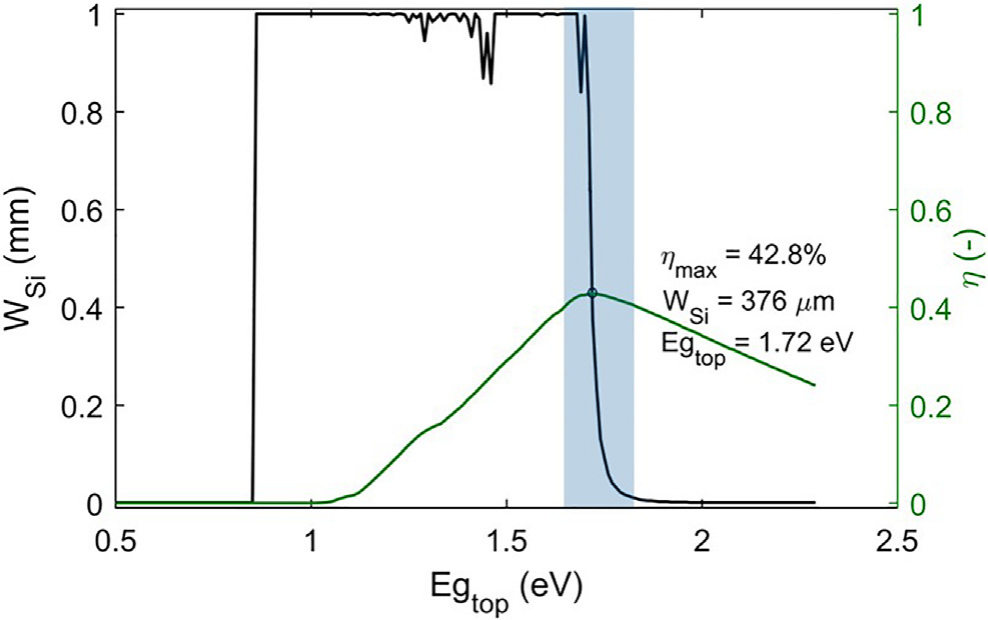
Silicon bottom cell bulk thickness (n-type) and tandem cell efficiency as a function of the band gap The efficiency limit and the Si thickness and top cell band gap at maximum efficiency are also mentioned. The blue area shows the band-gap range where top and bottom cell parameters, respectively band gap and thickness, are highly interdependent in determining the maximum efficiency. Therefore, the smoothness of the graph, which is linked to the voltage steps in the simulation code, is critical here.

The third example outcome is the maximum efficiency of the X-on-Si 2 terminal tandem cell and the associated cell parameters such as the band gap of the top cell and the thickness of the silicon bottom cell. At the STC condition, it is expected from the code that the maximum tandem cell efficiency with the value of 0.42789 happens at 1.72 eV of the top cell band gap and the silicon bottom cell thickness of 376 μm Figure 5 below shows an expected graph from the tandem cell simulation code which plots the interplay between the top cell bandgap and silicon bottom cell thickness to achieve the maximum efficiency. The blue highlighted region is highly sensitive to voltage steps in the simulation while the maximum efficiency happens in the same region. The smaller the voltage steps, the smoother and the more accurate the graph.

**Figure 6:**
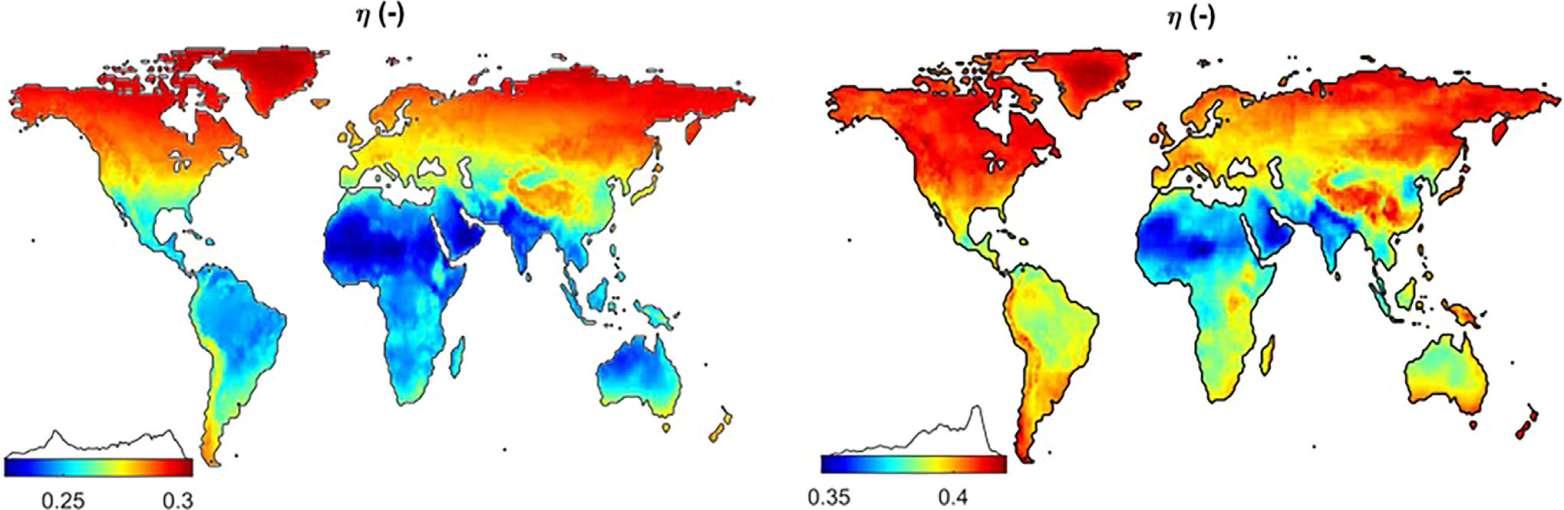
Achievable efficiency of mono-facial single junction silicon cell (left) and silicon-based double-junction 2-terminal tandem cell (right) visualized in the form of heat maps via the Map maker script

The fourth example of the expected outcome is solar cell simulation results, such as geographically dependent performance and optimum design parameters, in the form of global maps. Here the achievable global efficiency maps for single and double-junction silicon-based solar cells are plotted in Figure 6. For more maps, the reader is referred to the H. Ziar.^1^

## Limitations

MATLAB is not a free open-source software package that can impose an initial limitation on the protocol. However, most of the academia and industry have access to MATLAB using corporate accounts. Besides, thanks to the simplicity of the simulations, they can be conveniently transferred to other coding software packages that are free such as Python. Another potential limitation is the use of SMARTS which is mostly used for clear sky conditions. However, a combination of SMARTS output with an irradiance decomposition model, here the BRL model, makes it more reliable for other sky conditions as well. A further potential limitation is the high simulation time demand for many geographical locations. However, for single or limited input ambient conditions (irradiance and temperature), for instance, at standard test conditions, the protocol is very fast. The present protocol is based on highly cited publications and the most reliable physical and semi-conductor models present in the photovoltaics and solar fields. However, there could always be more advances in scientific findings that further improve the provided codes.

## Troubleshooting

The following issues might arise when implementing the protocol provided in this work.

### Problem 1

High simulation time when running for a large number of geographical locations at steps 12 and 13.

### Potential solution

Several simulation and technical approaches can be used to make the simulation faster. Using high power PC, using several normal PCs in parallel, pre-allocating memory, using parfor and other parallel computing techniques in MATLAB can speed up the geographical simulation. Besides, SMARTS original code which is in Fortran can be translated to MATLAB or Python so that the sunlight spectral irradiance simulation can be done within MATLAB, which eliminates the need for calling SMARTS, execution, and moving data. This will save time. Further, for the solar cell simulation codes, tuning the voltage steps and only running the code at low doping concentrations will reduce the simulation time.

### Problem 2

Distortion in the output curves, especially excess carrier concentration (***Δn***), carrier lifetime in silicon bulk (***τ***), and fill factor (***FF***), might happen sometimes at step 12.

### Potential solution

This is due to relatively large voltage steps (Vstep in the codes) sweeping over voltage values from zero to the band gap value. The solution is to make the voltage steps very small. Note that, the smaller the steps, the higher the simulation time though.

### Problem 3

A couple of times, the author encountered complex numbers for intrinsic recombination rates (***R***_***intr***_) yielded from the simulation at step 12.

### Potential solution

This is because excess carrier concentration (***Δn***) for very low voltage values obtains negative numbers and this will cause complex values for intrinsic recombination rates. These are unreadable values that could be skipped. A solution, however, is to only extract the real part of the number and allow the simulation to continue. This is important especially when a long simulation is planned and a sudden error or stop is undesirable. This is mentioned in the **Single-junction crystalline silicon solar cell simulation script** as a comment as well.

### Problem 4

The author also encountered one time an abnormally large aerosol optical depth (AOD) in the CERES dataset that caused the SMARTS software to abort. SMARTS considers a maximum of 5 for the AOD at 550 μm. Values higher than that will cause SMARTS to stop. This potential problem is related to the steps 5 and 11.

### Potential solution

It was most probably a faulty data recording. This was spotted with rather a timely investigation of AOD data, and that data point was replaced by the average of the recorded data from the previous and the next timestamp.

## Resource availability

### Lead contact

For further information and requests for resources and code availability, please contact Dr. Hesan Ziar (h.ziar@tudelft.nl).

### Technical contact

Technical questions on executing this protocol should be directed to and will be answered by the technical contact, Dr. Hesan Ziar (h.ziar@tudelft.nl).

### Materials availability

This study did not generate any new reagents.

### Data and code availability

All the MATLAB codes for this study can be regenerated by copy-pasting the scripts and functions provided in the paper and supplemental information. The datasets used in these MATLAB scripts and functions have a total size of 1.28 GB and are available at https://data.mendeley.com/ with a reserved https://doi.org/10.17632/867xkxhxy7.1. It is under embargo until 18-03-2025.

## Acknowledgments

The author would like to thank Tanja Post and Cas de Mooij for their efforts in identifying and retrieving data as well as scripting the first version of the SMARTS importer and parser codes. The author also appreciates the efforts put in by Sandeep Mishra and Tim Stark for preparations of the first and second versions of the BRL decomposition code. Table 3 mentions the names of individuals who worked on the versions of the MATLAB codes.

## Author contributions

H.Z.: writing – review and editing, writing – original draft, visualization, validation, supervision, software, resources, methodology, investigation, formal analysis, data curation, and conceptualization.

## Declaration of interests

The author declares no competing interests.

## References

[1] Ziar H. (2024). A global statistical assessment of designing silicon-based solar cells for geographical markets. Joule.

[2] Rodell M., Houser P.R., Jambor U., Gottschalck J., Mitchell K., Meng C.-J., Arsenault K., Cosgrove B., Radakovich J., Bosilovich M. (2004). The global land data assimilation system. Bull. Am. Meteorol. Soc..

[3] Gueymard C.A. (2001). Parameterized transmittance model for direct beam and circumsolar spectral irradiance. Sol. Energy.

[4] Gueymard C. (1995).

[5] Ridley B., Boland J., Lauret P. (2010). Modelling of diffuse solar fraction with multiple predictors. Renew. Energy.

[6] Gueymard C. (2006). Solar Conference.

[7] Brune W.H. (2024). Fundamentals of Atmospheric Science, Course lecture on "Ways to Specify Water Vapor". College of Earth and Mineral Sciences, The Pennsylvania State University. https://www.e-education.psu.edu/meteo300/node/519.

[8] (2024). Water Vapor in Air (course material). The University of Arizona. http://www.atmo.arizona.edu/students/courselinks/spring08/atmo336s1/courses/fall13/atmo551a/Site/ATMO_451a_551a_files/WaterVapor.pdf.

[9] (2024). Atmospheric Thermodynamics (lecture notes). University of Colorado. https://atoc.colorado.edu/%7Ecassano/atoc5050/Lecture_Notes/wh_ch3_part4.pdf.

[10] Wallace J.M., Hobbs P.V. (2006). Atmospheric Science: An Introductory Survey 92.

[11] Wielicki B.A., Barkstrom B.R., Harrison E.F., Lee R.B., Louis Smith G., Cooper J.E. (1996). Clouds and the Earth's Radiant Energy System (CERES): An earth observing system experiment. Bull. Am. Meteorol. Soc..

[12] Ziar H., Sönmez F.F., Isabella O., Zeman M. (2019). A comprehensive albedo model for solar energy applications: Geometric spectral albedo. Appl. Energy.

[13] Shockley W., Queisser H.J. (1961). Detailed balance limit of efficiency of p-n junction solar cells. J. Appl. Phys..

[14] Arora N.D., Hauser J.R., Roulston D. (1982). Electron and hole mobilities in silicon as a function of concentration and temperature. IEEE Trans. Electron Devices.

[15] Green M.A. (1984). Limits on the open-circuit voltage and efficiency of silicon solar cells imposed by intrinsic Auger processes. IEEE Trans. Electron Devices.

[16] Green M.A. (1990). Intrinsic concentration, effective densities of states, and effective mass in silicon. J. Appl. Phys..

[17] Misiakos K., Tsamakis D. (1993). Accurate measurements of the silicon intrinsic carrier density from 78 to 340 K. J. Appl. Phys..

[18] Schenk A. (1998). Finite-temperature full random-phase approximation model of band gap narrowing for silicon device simulation. J. Appl. Phys..

[19] Trupke T., Green M.A., Würfel P., Altermatt P.P., Wang A., Zhao J., Corkish R. (2003). Temperature dependence of the radiative recombination coefficient of intrinsic crystalline silicon. J. Appl. Phys..

[20] Altermatt P.P., Geelhaar F., Trupke T., Dai X., Neisser A., Daub E. (2005). NUSOD’05. Proceedings of the 5th International Conference on Numerical Simulation of Optoelectronic Devices.

[21] Altermatt P., Schenk A., Schmithüsen B., Heiser G. (2006). A simulation model for the density of states and for incomplete ionization in crystalline silicon. II. Investigation of Si:As and Si:B and usage in device simulation. J. Appl. Phys..

[22] Green M.A. (2008). Self-consistent optical parameters of intrinsic silicon at 300 K including temperature coefficients. Sol. Energy Mater. Sol. Cell..

[23] McIntosh K.R., Altermatt P.P. (2010). 2010 35th IEEE Photovoltaic Specialists Conference.

[24] Richter A., Glunz S.W., Werner F., Schmidt J., Cuevas A. (2012). Improved quantitative description of Auger recombination in crystalline silicon. Phys. Rev. B.

[25] Rüdiger M., Greulich J., Richter A., Hermle M. (2013). Parameterization of free carrier absorption in highly doped silicon for solar cells. IEEE Trans. Electron. Dev..

[26] Richter A., Hermle M., Glunz S.W. (2013). Reassessment of the limiting efficiency for crystalline silicon solar cells. IEEE J. Photovolt..

[27] Engelbrecht D.A., Tiedje T. (2021). Temperature and intensity dependence of the limiting efficiency of silicon solar cells. IEEE J. Photovolt..

[28] Rühle S. (2016). Tabulated values of the Shockley–Queisser limit for single junction solar cells. Sol. Energy.

