# Supplementary material for: Protocol to simulate crystalline Si-based single- and multi-junction solar cells under standard test and real-world conditions via MATLAB scripts
Source: STAR Protoc. 2024 Dec 3;5(4):103464. doi: 10.1016/j.xpro.2024.103464 (PMC11653140; doi:10.1016/j.xpro.2024.103464)
Supplement: Document S1. Methods S1–S17 [file mmc1.pdf]

Methods S1: SMARTS parser function loads atmospheric data, runs SMARTS and saves outputs, related to Step 11.

```
function SMARTS_parser(filename1, year, month, day, hour, zone, co_min, co_max)
%% reshape time input
time=[]; day_input=[]; month_input=[]; day_nr = (1:1:365);
%% prealocating
dmax=zeros(1,12);  time1=zeros(1,24); day1=zeros(1,24); month1=zeros(1,12);
for m = 1:length(month)
    if m==2                                 % February
        dmax(m)=28;
    elseif m==4 || m==6 || m==9 || m==11     % April, June, September, November
        dmax(m)=30;
    else
        dmax(m)=31;
    end
    for d=1:dmax(m)
        for h=1:length(hour)                 % Time zone not included
            time1(h) = hour(h); day1(h) = day(d); month1(h) = month(m);
         end
        time2=reshape(time1,24,[]); time = cat(2,time,time2);
        day2=day1(2:24:end); day_input = cat(2,day_input,day2);
        month2=month1(2:24:end); month_input = cat(2,month_input,month2);
     end
end
%% Loading input data prepared at major step one    % in supplementary dataset
addpath 'C:\DirectoryOfTheProjectData\Input_for_Smarts'
load('Surf_Press_24h_2019_input.mat', 'Surf_Press_24h_2019_input')
load('prec_water_24h_2019_input.mat','prec_water_24h_2019_input')
load('ozone_24h_2019_input.mat','ozone_24h_2019_input')
load('AOD_550_24h_2019_input.mat','AOD_550_24h_2019_input')
load('Tair_C_24h_2019_input.mat','Tair_C_24h_2019_input')
load('Tair_C_Daily_2019_input.mat','Tair_C_Daily_2019_input')
load('Rel_Humi_24h_2019_input.mat','Rel_Humi_24h_2019_input')
load('Altit_input.mat','Altit_input')
load('Urban_Extents_input.mat','Urban_Extents_input')
load('Snow_24h_2019_input.mat','Snow_24h_2019_input')
load('Land_coord.mat','Land_coord')
%% Filling in the Cards
% The directory is adjusted per user:
project_path = 'C:\DirectoryOfTheProjectData\Repository';
% This is the path were you have an installation of SMARTS2.9.5
SMARTS_path = 'C:\SMARTSInstallationDirectory';
Winter = [char(39) 'Winter' char(39)]; Summer = [char(39) 'Summer' char(39)];
URBAN = [char(39) 'S' char(38) 'F_URBAN' char(39)];
RURAL = [char(39) 'S' char(38) 'F_RURAL' char(39)];
for co=co_min:co_max
    for t=1:length(day_nr)
        fprintf('%.1f %.1f location %d of %d t=%d of %d\n', Land_coord(co,1),
Land_coord(co,2), co, length(Land_coord), t, length(day_nr))
       current_folder = pwd;                     % identifies current folder
    if strcmp(current_folder, project_path) ~= 1     % compares project path
 % with the current folder
        cd(project_path)
    end
```

```
    cd('SMARTS_input\'); copyfile(filename1, 'tmp.txt');
    fidr = fopen('tmp.txt','r');                          % open for reading
    Card = textscan(fidr,'%s','delimiter','\n');
    fclose(fidr);

    Card{1,1}{3,1}=sprintf('%.2f %.2f %d',
Surf_Press_24h_2019_input(co,t),Altit_input(co),0);   % surface pressure
                                                       % CERES data & altitude
    if Land_coord(co,1)>0                              % north
        if day_nr(t)<=59 || day_nr(t)>=244         % 28 Feb>= date, 1 Sept<=date
                                                   % (meteorological fall+winter)
            Card{1,1}{5,1}=sprintf('%.2f %.2f %s %.2f',
Tair_C_24h_2019_input(co,t),Rel_Humi_24h_2019_input(co,t),Winter,
Tair_C_Daily_2019_input(co,day_nr(t)));     % humidity & temperature, GLDAS data
        else                                % (meteorological spring+summer)
            Card{1,1}{5,1}=sprintf('%.2f %.2f %s %.2f',
Tair_C_24h_2019_input(co,t),Rel_Humi_24h_2019_input(co,t),Summer,
Tair_C_Daily_2019_input(co,day_nr(t)));     % humidity & temperature, GLDAS data
        end
    else                                        % south
        if day_nr(t)<=59 || day_nr(t)>=244  % 28 Feb>= date, 1 Sept<=date
                                            % (meteorological spring+summer)
            Card{1,1}{5,1}=sprintf('%.2f %.2f %s %.2f',
Tair_C_24h_2019_input(co,t),Rel_Humi_24h_2019_input(co,t),Summer,
Tair_C_Daily_2019_input(co,day_nr(t)));     % humidity & temperature, GLDAS data
        else                                % (meteorological fall+winter)
            Card{1,1}{5,1}=sprintf('%.2f %.2f %s %.2f',
Tair_C_24h_2019_input(co,t),Rel_Humi_24h_2019_input(co,t),Winter,
Tair_C_Daily_2019_input(co,day_nr(t)));     % humidity & temperature, GLDAS data
        end
    end
    Card{1,1}{7,1}=sprintf('%.2f',prec_water_24h_2019_input(co,t));
                                              % precipitable water, CERES data
Card{1,1}{9,1}=sprintf('%.f %.2f',1,ozone_24h_2019_input(co,t)); % O3 column
from CERES data
    if Urban_Extents_input(co)==2
        Card{1,1}{13,1}=sprintf('%s', URBAN);
    else                                      %'S&F_RURAL' (default)
    Card{1,1}{13,1}=sprintf('%s', RURAL);
    end
Card{1,1}{15,1}=sprintf('%.3f',AOD_550_24h_2019_input(co,t)); % AOD @ 550 nm
    if Snow_24h_2019_input(co,1)>=50 && Land_coord(co,1) > 0  % snow & northern
hemisphere(default)
        Card{1,1}{16,1}=sprintf('%d',3); % snow
        Card{1,1}{18,1}=sprintf('%d %.2f %d',3,abs(Land_coord(co,1)),180); %tilt
angle = latitude
    elseif Snow_24h_2019_input(co,1)>=50  % snow & southern hemisphere
        Card{1,1}{16,1}=sprintf('%d',3);  % snow
        Card{1,1}{18,1}=sprintf('%d %.2f %d',3,abs(Land_coord(co,1)),0); %tilt
angle = latitude
    elseif Land_coord(co,1) > 0  % light soil(38, default) & northern hemisphere
        Card{1,1}{16,1}=sprintf('%d',38);                    % light soil
        Card{1,1}{18,1}=sprintf('%d %.2f %d',38,abs(Land_coord(co,1)),180);
%tilt angle = latitude
```

```
    else                          % light soil(38, default)& southern hemisphere
        Card{1,1}{16,1}=sprintf('%d',38);                     % light soil
        Card{1,1}{18,1}=sprintf('%d %.2f %d',38,abs(Land_coord(co,1)),0);  %tilt
angle = latitude
    end
fidw = fopen('tmp.txt','wt') ;    % opens the .txt file for writing
    fprintf(fidw,'%s\n',Card{1,1}{:}); % overwrite txt with altered data
    fclose(fidw);
    fid = fopen('tmp.txt', 'a+'); % Append data to the end of the .txt file
    % Here we add all the hours of day to the last card of SMARTS:
    for u=1:24
    s = sprintf('%d %d %d %.2f %.2f %.2f %.f', year, month_input(t),
day_input(t), time(u,t), Land_coord(co,1), Land_coord(co,2), zone);
    fprintf(fid, '%s\r\n', s);
    end
    fclose all;

movefile('tmp.txt', [SMARTS_path '\smarts295.inp.txt'], 'f');
cd(SMARTS_path); command = 'smarts295bat.exe'; system(command); % It runs SMARTS
% The .txt file names are adjusted per user:
movefile('smarts295.ext.txt', [project_path
'\SMARTS_output\SMARTS_output_ext\Irr_2019_24h.ext.txt'])
movefile('smarts295.out.txt', [project_path
'\SMARTS_output\SMARTS_output_out\Irr_2019_24h.out.txt'])
movefile('smarts295.inp.txt', [project_path
'\SMARTS_input\last_input\last_input_24h.txt'])

save_ext_here = 'C:\DirectoryOfTheProjectData\Repository\SMARTS_OUTPUT_SAVE';
save_out_here = 'C: \DirectoryOfTheProjectData\Repository\SMARTS_OUTPUT_SAVE';

cd(project_path); cd('SMARTS_output\SMARTS_output_ext\');
input_ext = dir( fullfile('Irr_2019_24h.ext.txt') );            % rename
ext_name_old = { input_ext.name };
inputFullFileName = fullfile(pwd, ext_name_old{1:1});
outputBaseFileName = sprintf('%s_%02.0f_%03.0f_%04.0f.ext.txt',
ext_name_old{1:1}(1:end-8), Land_coord(co,1), Land_coord(co,2), day_nr(t));
outputFullFileName = fullfile(save_ext_here, outputBaseFileName);
movefile(inputFullFileName, outputFullFileName);

cd(project_path); cd('SMARTS_output\SMARTS_output_out\');
input_ext2 = dir( fullfile('Irr_2019_24h.out.txt') );           % rename
ext_name_old2 = { input_ext2.name };
inputFullFileName2 = fullfile(pwd, ext_name_old2{1:1});
outputBaseFileName2 = sprintf('%s_%02.0f_%03.0f_%04.0f.out.txt',
ext_name_old2{1:1}(1:end-8), Land_coord(co,1), Land_coord(co,2), day_nr(t));
% (1:end-8) to remove .out.txt of the old file name
outputFullFileName2 = fullfile(save_out_here, outputBaseFileName2);
movefile(inputFullFileName2, outputFullFileName2);
  end
end
end
```

Methods S2: Auxiliary commands to fill in SMARTS Input Cards numbered: 4, 6, 8, 10-12, 14, 17, 19-29 that are the same for all coordinates, related to Step 11.

```
%     Card{1,1}{4,1}= sprintf('%d',0);  % To have non-reference atmosphere in SMARTS, this row should be zero
%     Card{1,1}{6,1}= sprintf('%d',0);  % To specify precipitable water in SMARTS, this row should be zero
%     Card{1,1}{8,1}= sprintf('%d',0);  % To specify Ozon vector column in SMARTS, this row should be zero
%     Card{1,1}{10,1}= sprintf('%d',1);  % To use default method for gaseous absorption and pollution calcuations, this row should be 1.
%     Card{1,1}{11,1}= sprintf('%.2f',411.66);  % Average CO2 concentration for year 2019.
%     Card{1,1}{12,1}= sprintf('%d',1);  % To use the default extraterrestrial spectrum (Gueymard 2002), this row should be 1.
%     Card{1,1}{14,1}= sprintf('%d',5);  % To specify that the input AOD is at 550 nm, this row should be 5.
%     Card{1,1}{17,1}= sprintf('%d',1);  % To specify that the input for albedo is NOT bypassed in SMARTS.
%     Card{1,1}{19,1}=sprintf('%d %d %d %d',280, 4000, 1, 1367); % To specify spectral range, solar constant, and solar constant distant correction factor in SMARTS
%     Card{1,1}{20,1}=sprintf('%d',2); % To specify type of output files, printed spectral range, and spectral steps
%     Card{1,1}{21,1}=sprintf('%d %d %.f',280, 4000, 0.5); % To Specify printed spectral range and spectral step
%     Card{1,1}{22,1}=sprintf('%d',3); % To Specify number of output spectra
%     Card{1,1}{23,1}=sprintf('%d %d %d',4,6,7);  % To Specify type of output spectra (GHI, direct tilted, and diffuse tilted irradiances)
%     Card{1,1}{24,1}=sprintf('%d',1);  % To Specify circumsolar calculations are NOT bypassed in SMARTS
%     Card{1,1}{25,1}=sprintf('%d %.f %d',0, 2.9, 0); % To specify radiometer geometry (slope, aperture, and limit)
%     Card{1,1}{26,1}=sprintf('%d',0); % To bypass smoothing filter in SMARTS
%     Card{1,1}{27,1}=sprintf('%d',0); % To bypass PAR calculations in SMARTS
%     Card{1,1}{28,1}=sprintf('%d',0); % To bypass extra UV calculate. in SMARTS
%     Card{1,1}{29,1}=sprintf('%d',3); % To specify type of solar position and airmass calculations
```

Methods S3: SMARTS importer function extracts data from SMARTS output files and creates workspace variables for MATLAB, related to Step 11.

```
function [SMARTS_irr_clear_sky_area, wvlngth, L, hours] =
SMARTS_importer(Land_coord, project_path, days_2019, co_min, co_max)
time=[]; day_nr = (1:1:365);
for a=1:365
time2=1:24; time = cat(2,time,time2);
end
cd('C:\ DirectoryOfTheProjectData\Repository\SMARTS_OUTPUT_SAVE')
NameBase=('Irr_2019_24h'); a=1;
outputName = cell(1,(co_max-co_min+1)*length(days_2019));  % prealocating cell
output_files = struct('name', cell(1,(co_max-co_min+1)*length(days_2019)),
'folder', cell(1,(co_max-co_min+1)*length(days_2019)), 'date', cell(1, (co_max-
co_min+1)*length(days_2019)), ...
                      'bytes', cell(1,(co_max-co_min+1)*length(days_2019)),
'isdir', cell(1,(co_max-co_min+1)*length(days_2019)), 'datenum', cell(1,(co_max-
co_min+1)*length(days_2019)));                          % prealocating structure

for co=co_min:co_max                                        % length(Land_coord)
    fprintf('SMARTS importer, list of filenames, location %d of %d\n', co,
length(Land_coord))
        for t=1:length(days_2019)
        outputFileName = sprintf('%s_%02.0f_%03.0f_%04.0f.ext.txt', NameBase,
Land_coord(co,1), Land_coord(co,2), day_nr(t));
        outputName{1,a}={outputFileName};
        output_files(a) = dir( fullfile(outputName{1,a}{1,1}) ); a=a+1; end
end
clear a outputFileName outputName
filenames={output_files.name}'; sum_irr=cell(length(Land_coord),1); row=co_min;
for a=1:(length(filenames)/length(days_2019))               % number of locations
    fprintf('SMARTS importer, location %d of %d\n', a,
(length(filenames)/length(days_2019)))
  for b=1:length(days_2019)
     fprintf('SMARTS importer, location %d of %d, time %d of %d\n', a,
(length(filenames)/length(days_2019)), b, length(days_2019))
    raw_table = readtable([filenames{b+((a-1)*length(days_2019))}]); % creates a
table by reading column oriented data from a file
    vars = raw_table.Properties.VariableNames; % header of each new table
    raw_cell = table2cell(raw_table);          % Converts table to cell array
    if isempty(raw_cell) == 0
        clear raw_array
        A=raw_cell(:,1); B=raw_cell(:,2); C=raw_cell(:,3); D=raw_cell(:,4);
% When you add one more output to SMARTS, here you need to add another cell and
call it letter E.
        if isa(raw_cell{1,1},'char') == 1
            raw_array(:,1) = str2double(A); else
            raw_array(:,1) = cell2mat(A); end
       if isa(raw_cell{1,2},'char') == 1
           raw_array(:,2) = str2double(B); else
           raw_array(:,2) = cell2mat(B); end
       if isa(raw_cell{1,3},'char') == 1
           raw_array(:,3) = str2double(C); else
           raw_array(:,3) = cell2mat(C); end
```

```
        if isa(raw_cell{1,4},'char') == 1
            raw_array(:,4) = str2double(D); else
            raw_array(:,4) = cell2mat(D); end
% When you add one more output to SMARTS, here you need to add another "if-else" and call it letter E.
    else
        raw_array = cell2mat(raw_cell);
    end

if output_files(1,b+((a-1)*length(days_2019))).bytes > 0 % no need to run when file is empty
    [L, hours] = check_length(raw_array); % function at end of script, L=rows of one table hours=number of tables
    wvlngth = raw_array(1:L, 1);          % same for all locations
for k = 2:length(vars)
    irr.(vars{k}) = zeros(L,hours);
    for t = 1:hours
        if t == 1
            irr.(vars{k})(:,t) = raw_array(1:L,k); else
            irr.(vars{k})(:,t) = raw_array((((t-1)*L+(t-1))+1):((t*L+t)-1),k);
        end
    end
end
if exist('irr_together') == 0
irr_together.(vars(1))=[]; irr_together.(vars(1))=[]; irr_together.(vars{4})=[];
% When you add one more output to SMARTS, here you need to add another line >>> irr_together.(vars(1)) = []; end
     irr_together.(vars(1)) = cat(2,irr_together.(vars(1)),irr.(vars(1)));
     irr_together.(vars(1)) = cat(2,irr_together.(vars(1)),irr.(vars(1)));
     irr_together.(vars{4}) = cat(2,irr_together.(vars{4}),irr.(vars{4}));
% When you add one more output to SMARTS, here you need to add another line >>> irr_together.(vars(1))
    end
end
  if exist('sum_irr') == 0 % it can happen in the far north (or south) that the sun does not come above the horizon the whole day
      sum_irr{row} = []; else
      sum_irr{row} = irr_together; end      % writes the spectral data on
  row = row+1;
  clear irr_together
  end
function [L, t] = check_length(A)
        t = 1; L= length(A);
        for i = 1:length(A)
            if sum(isnan(A(:,1:2)) == true) ==0
                L= length(A); t= 1;
            elseif (isnan(A(i,1)) == true) && (t == 1)
                L = i - 1; t = t + 1;
            elseif isnan(A(i,1)) == true
                t = t + 1;
            end
        end
    end
```

```
SMARTS_irr_clear_sky_area=sum_irr;
filenames_importer=reshape(filenames,length(days_2019),[]);

cd(project_path)
save('SMARTS_irr_clear_sky.mat', 'SMARTS_irr_clear_sky_area', '-v7.3')
input_ext = dir( fullfile('SMARTS_irr_clear_sky.mat') );          % rename
ext_name_old = { input_ext.name };
inputFullFileName = fullfile(pwd, ext_name_old{1:1});
outputBaseFileName = sprintf('%s_%d-%d.mat', ext_name_old{1:1}(1:end-4), co_min,
co_max); %(1:end-4) to remove .txt of the old file name
outputFullFileName = fullfile(pwd, outputBaseFileName);
movefile(inputFullFileName, outputFullFileName);

save('filenames_importer.mat', 'filenames_importer')
end
```

Methods S4: SMARTS zenith importer function which extracts Sun zenith values from SMARTS output files, related to Step 11.

```
function [all_zenith_SMARTS, filenames_zen] = zenith_importer(Land_coord, project_path, days_2019, co_min, co_max)
cd('C:\DirectoryOfTheProjectData\Repository\SMARTS_OUTPUT_SAVE')
NameBase=('Irr_2019_24h'); a=1;
outputName = cell(1,(co_max-co_min+1)*length(days_2019));  % prealocating cell
output_files = struct('name', cell(1,(co_max-co_min+1)*length(days_2019)), 'folder', cell(1,(co_max-co_min+1)*length(days_2019)), 'date', cell(1, (co_max-co_min+1)*length(days_2019)), ...
                      'bytes', cell(1,(co_max-co_min+1)*length(days_2019)), 'isdir', cell(1,(co_max-co_min+1)*length(days_2019)), 'datenum', cell(1,(co_max-co_min+1)*length(days_2019)));                        % prealocating structure
for co=co_min:co_max
    fprintf('zenith importer, list of filenames, %d of %d\n', co, length(Land_coord))
        for t=1:length(days_2019)
        outputFileName = sprintf('%s_%02.0f_%03.0f_%04.0f.out.txt', NameBase, Land_coord(co,1), Land_coord(co,2), days_2019(t));
        outputName{1,a}={outputFileName};
        output_files(a) = dir( fullfile(outputName{1,a}{1,1})); a=a+1;
        end
end
clear outputFileName outputName

filenames_z = { output_files.name }; % the names of the txt files
sum_zenith=zeros((length(filenames_z)/length(days_2019)),length(days_2019)*24);
check={'Zenit =  90.00'};
for a=1:(length(filenames_z)/length(days_2019))           % number of locations
    tk4= [];
    fprintf('Location %d of %d\n', a, (length(filenames_z)/length(days_2019)))
  for b=1:length(days_2019)
    fid  = fopen(filenames_z{1,b+((a-1)*length(days_2019))},'r');
    text = textscan(fid,'%s','Delimiter','','endofline','');
    text = text{1}{1};
    fclose(fid);
    tk = regexp(text,'Zenit = \d*\.?\d+|Zenit =  \d*\.?\d+|Zenith Angle (apparent\D* \d*\.?\d+','match');
    tk2 = (regexp(tk,'[\d.]+','match'));
    tk3 = zeros(1,length(tk2));                           % prealocation
    for c=1:length(tk2)
        tk3(c) = sscanf(tk2{1,c}{1,1}, '%f');
        if length(tk{1,c})==length(check{1,1})
            compare=(tk{1,c}== check{1,1});
            if sum(compare)==14
                tk3(c)=tk3(c)+0.001; % there exist a few cases in which the irr is calculaed at a 90 deg angle (probably 89.999999), adding 0.001 to the not-simulated angles helps night filtering later on
            end
        end
        tk4 = cat(2,tk4,tk3(c));
     end
  end
  sum_zenith(a,:) = tk4;
end
```

```
 all_zenith_SMARTS=sum_zenith; filenames_zen=filenames_z;
cd(project_path)
save('all_zenith_SMARTS.mat', 'all_zenith_SMARTS')
save('filenames_zen.mat', 'filenames_zen')
end
```

Methods S5: MATLAB function for BRL decomposition model calculates the diffuse and direct ratios of global horizontal irradiance, related to Step 11.

```
function [df, dr] = BRL_model(Land_coord, month, hour, co_min, co_max)
addpath 'C:\DirectoryOfTheProjectData\nc files\CERES_BRL_input'
% in supplementary dataset
load('TOA_ini_1h_2019_input.mat','TOA_ini_1h_2019_input')
load('sfc_ini_1h_2019_input.mat','sfc_ini_1h_2019_input')
load('CERES_zenith_1h_2019_input.mat','CERES_zenith_1h_2019_input')

Global = sfc_ini_1h_2019_input; H0=TOA_ini_1h_2019_input;
theta_z=CERES_zenith_1h_2019_input;
clear sfc_ini_24h_2019_input TOA_ini_24h_2019_input CERES_zenith_24h_2019_input

Size=[size(Global,1),1];
EQT = cell(Size); AST = cell(Size); omega = cell(Size); delta = cell(Size);
alpha = cell(Size); cos_omega_s = cell(Size); omega_sunrise = cell(Size);
omega_sunset = cell(Size); Kt = cell(Size); Persistence = cell(Size);
df = cell(Size); Idif_Wm = cell(Size); Idir_Wm = cell(Size); dr = cell(Size);
days=365; time=[]; time1 = zeros(1,24);                    % prealocating

for m = 1:length(month)
    if m==2                                       % February
        dmax=28;
    elseif m==4 || m==6 || m==9 || m==11          % April, June, September, November
        dmax=30;
    else
        dmax=31;
    end
    for d=1:dmax
        for h=1:length(hour)
            time1(h) = hour(h);                   % Time zone not included
        end
        time = cat(2,time,time1);
    end
end
one_day=ones(1,24); doy = zeros (1,8760);     % prealocating
for i=1:365
    doy((1+(24*(i-1)):24+(24*(i-1))))=one_day*i;
end
clear hour time1 doy1
LH = time-0.5;
%% Determine time zones
Timezone_boarders(:,1)=[-180 -180+7.5:15:180-7.5 180];
Timezone_boarders(:,2)=[0 -12:1:12]; TZ=zeros(length(Land_coord(:,1)),1);
for x=1:length(Land_coord(:,2))
   f=find(Land_coord(x,2)<Timezone_boarders(:,1),1);
   TZ(x,1)=Timezone_boarders(f,2);            % Time Zone (relative to UTC)
end

for co=co_min:co_max
%% Calculate apparent solar time AST
EQT{co} = ones(length(time),1);               % vector for equation of time
AST{co} = ones(length(time),1);               % vector for apparent solar time
gamma = zeros (1,length(time));               % prealocating
```

```
for i = 1:length(time)
    gamma(i) = 2.*pi.*((doy(i)-1)/365);       % day angle
    EQT{co}(i) = (0.000075 + (0.001868*cos(gamma(i))) - (0.032077*sin(gamma(i)))
- (0.014615*cos(2*gamma(i))) - (0.04089*sin(2*gamma(i)))) * 229.18; % Equation
of Time
    AST{co}(i) = LH(i) + TZ(co,1) - Land_coord(co,2) / 15 + EQT{co}(i) / 60;
end

%% clearness index
omega{co} = ones(length(time),1);             % hour angle
delta{co} = ones(length(time),1);             % solar declination
alpha{co} = ones(length(time),1);             % solar altitude
kt = cell(1,co_max);                          % prealocating
for i = 1:length(time)
    gamma(i) = 2.*pi.*((doy(i)-1)/365); %day angle
    delta{co}(i) = (0.006918 - (0.399912.*cos(gamma(i))) +
(0.070257.*sin(gamma(i))) - (0.006758.*cos(2*gamma(i))) +
(0.000907.*sin(2*gamma(i))) - (0.002697.*cos(3*gamma(i))) +
(0.00148.*sin(3*gamma(i))))*(180/pi); %declination angle
    omega{co}(i) = (LH(i) + TZ(co,1) - 12) * (-15);        % hour angle
    alpha{co}(i) = 90 - theta_z(co,i);                     % sun's altitude
    kt{co}(i) = Global(co,i)/H0(co,i);                     % clearness index
    if kt{co}(i) < 0
        kt{co}(i) = 0;
    elseif kt{co}(i) > 1
        kt{co}(i) = 1; Global(co,i) = round(H0(co,i)); end
end
kt{co}(isnan(kt{co}))=0;                                   % when glo/H0=0/0
cos_omega_s{co}=ones(length(time),1);
omega_sunrise{co}=ones(length(time),1); omega_sunset{co}=ones(length(time),1);

for i=1:length(time)
   cos_omega_s{co}(i) = -tand(Land_coord(co,1))*tand(delta{co}(i));
   omega_sunrise{co}(i) = -acosd(cos_omega_s{co}(i));
   omega_sunset{co}(i) = acosd(cos_omega_s{co}(i));
end
%% daily clearness index
Kt{co} = ones(length(time),1);
for i = 1:days
    GlobSum = 0; HSum = 0;
    for j=1:24
       if alpha{co}((i-1)*24+j) > 10 && Global(co,((i-1)*24+j))>=0 &&
~isnan(Global(co,((i-1)*24+j)))
           GlobSum = GlobSum + Global(co,((i-1)*24+j));
           HSum = HSum + H0(co,((i-1)*24+j)); end
    end
    if GlobSum == 0                  % calculate again, include values alpha<10
        for j=1:24
            if Global(co,((i-1)*24+j))>=0 && ~isnan(Global(co,((i-1)*24+j)))
                GlobSum = GlobSum + Global(co,((i-1)*24+j));
                HSum = HSum + H0(co,((i-1)*24+j)); end
        end
    end
```

```
    for j = 1:24
        Kt{co}((i-1)*24+j) = GlobSum/HSum;
    end
end

%% Persistence
Persistence{co} = ones(length(time),1);
for i = 2:length(time)-1
   if omega_sunrise{co}(i) < omega{co}(i) && omega_sunrise{co}(i) > omega{co}(i-
1) && kt{co}(i) > 0
       Persistence{co}(i) = kt{co}(i);
   elseif omega_sunset{co}(i) > omega{co}(i) && omega_sunset{co}(i) <
omega{co}(i+1) && kt{co}(i) > 0
       Persistence{co}(i) = kt{co}(i);
   elseif kt{co}(i-1) > 0 && kt{co}(i+1) > 0 && kt{co}(i) >0
       Persistence{co}(i) = (kt{co}(i-1)+kt{co}(i+1))/2;
   else
       Persistence{co}(i) = 0;
   end
end

%% diff fraction d, BRL formula + I_dif calculation + I_dif filter
df1 = cell(1,co_max); df1{co} = ones(length(time),1);
Idif_Wm{co} = ones(length(time),1); Idir_Wm{co} = ones(length(time),1);
dr{co} = ones(length(time),1);

for i=1:length(time)
   df1{co}(i) = 1/(1+exp(-5.38+6.63*kt{co}(i)+0.006*AST{co}(i)-
0.0077*alpha{co}(i)+1.75*Kt{co}(i)+1.31*Persistence{co}(i)));
   Idif_Wm{co}(i) = df1{co}(i) * Global(co,i);
   if Idif_Wm{co}(i) > Global(co,i)
           Idif_Wm{co}(i) = Global(co,i); end
   if Idif_Wm{co}(i) < 0
       Idif_Wm{co}(i) = 0; end
   df{co}(i,1)=Idif_Wm{co}(i)/Global(co,i);  % DNI/GHI for HORIZONTAL surface
   Idir_Wm{co}(i)=(Global(co,i)-Idif_Wm{co}(i))/cos(deg2rad(theta_z(co,i)));
                                             % GHI = DNI*cos(theta) + DHI
   dr{co}(i) = Idir_Wm{co}(i)/Global(co,i);  % DNI/GHI
end
end
save('df.mat', 'df'); save('dr.mat', 'dr');
end
```

Methods S6: Night filter function filters out nighttime data, related to Step 11.

```
function [input_df, input_dr, SMARTS_zenith, Time_day] =
Night_filter_function(Total_time, all_zenith_SMARTS, df, dr, project_path,
co_min, ~)
Size=length(df); input_df = cell(Size,1); input_dr = cell(Size,1);
SMARTS_zenith = cell(Size,1); T_K_input_avg = cell(Size,1);
T_K_input = cell(Size,1);
addpath 'C:\DirectoryOfTheProjectData\nc files\GLDAS_nc-files'
load('T_K_3h_2019_input.mat','T_K_3h_2019_input')    % in supplementary material
T_K_timer = zeros(length(all_zenith_SMARTS),1); a3=1;      % prealocating
for a1=1:length(all_zenith_SMARTS(1,:))/3
    for a2=1:3
        T_K_timer(a3,1)=a1; a3=a3+1;
    end
end
clear a1 a2 a3
co = co_min;
fprintf('Night filter\n')
for a= 1:length(all_zenith_SMARTS(:,1))  % all_zenith_SMARTS(:,1) has the same
size as co_max-co_min
    c=1;
    for x= 1:length(all_zenith_SMARTS(1,:))
        if all_zenith_SMARTS(a,x)<=90 % Some 90deg angles have been used in
simulations (probably 89.9999),to not-simulated 90deg angles 0.001 has been
added
            input_df{co}(c,1)=df{co}(x); input_dr{co}(c,1)=dr{co}(x);
            SMARTS_zenith{co}(c,1)=all_zenith_SMARTS(a,x);
            T_K_input1{a,1}(c,1)=T_K_3h_2019_input(co,T_K_timer(x));
            Time_day{a}(1,c)=Total_time(x); c=c+1;
        else                            % thus zenith>90 (sun below horizon)
                                        % do nothing
        end
    end
   co=co+1;
end
co = co_min;
for a=1:length(T_K_input1)
    t1=1;
    for c=1:length(T_K_input1{a,1})
        if ~isnan(input_dr{co,1}(c,1))
        T_K_input{co,1}(t1,1)=T_K_input1{a,1}(c,1); t1=t1+1;
        else
        end
    end
    T_K_input_avg{co,1}=mean(T_K_input{co, 1});
    co=co+1;
end
cd(project_path); save('input_df.mat', 'input_df');
save('input_dr.mat', 'input_dr'); save('SMARTS_zenith.mat', 'SMARTS_zenith')
save('T_K_input.mat', 'T_K_input'); save('T_K_input_avg.mat', 'T_K_input_avg')
end
```

Methods S7: BRL ratios function calculates the spectral share of direct and diffuse components of irradiance on a target tilted plane, related to Step 11.

```
function[G_direct_1h_2019_all_BRL, G_diffuse_1h_2019_all_BRL,
G_POA_1h_2019_all_BRL] = BRL_ratios(SMARTS_zenith, input_df, input_dr, co_min,
co_max)
fprintf('loading..\n')                       % Building a correct loading name
load(fullfile(sprintf('%s_%d-%d.mat', 'SMARTS_irr_clear_sky',co_min,co_max)));
load('Land_coord.mat','Land_coord')          % in supplementary material
%% Share of Direct and Diffuse components (G_direct and G_diffuse) from SMARTS
addpath 'C:\DirectoryOfTheProjectData'
load('wvlngth.mat')
G_direct_1h_2019_hourly_avg = cell(co_max,1);   % prealocating
G_diffuse_1h_2019_hourly_avg = cell(co_max,1);  % prealocating
for co= co_min:co_max
    for c= 1:size(SMARTS_irr_clear_sky_area{co,1}.Direct_tilted_irradiance,2)
    G_direct_1h_2019_hourly_avg{co,1}(1,c) = trapz
(wvlngth,SMARTS_irr_clear_sky_area{co,1}.Direct_tilted_irradiance(:,c));
    G_diffuse_1h_2019_hourly_avg{co,1}(1,c) = trapz
(wvlngth,SMARTS_irr_clear_sky_area{co,1}.Difuse_tilted_irradiance(:,c));
    end
end
SMARTS_dr_tilted = cell (co_max,1); SMARTS_df_tilted = cell (co_max,1);
for co= co_min:co_max
SMARTS_dr_tilted{co,1}=G_direct_1h_2019_hourly_avg{co,1}./(G_diffuse_1h_2019_hou
rly_avg{co,1} + G_direct_1h_2019_hourly_avg{co,1});
SMARTS_df_tilted{co,1}=G_diffuse_1h_2019_hourly_avg{co,1}./(G_diffuse_1h_2019_ho
urly_avg{co,1} + G_direct_1h_2019_hourly_avg{co,1});
end
%% Finding G_direct and G_diffuse with and without considering BRL shares for
tilted surface. Also calculating GHI for horizontal surface from SMARTS
G_direct_1h_2019_all_BRL = cell(co_max,1);          % prealocating
G_diffuse_1h_2019_all_BRL = cell(co_max,1);         % prealocating
G_direct_1h_2019_all_SMARTS = cell(co_max,1);       % prealocating
G_diffuse_1h_2019_all_SMARTS = cell(co_max,1);      % prealocating
GHI_1h_2019_all_SMARTS = cell(co_max,1);            % prealocating
input_dr_tilted = cell(co_max,1);  input_df_tilted = cell(co_max,1);
mean_zenith = cell(co_max,1);                       % prealocating
for a= co_min:co_max %a= 1:length(SMARTS_irr_cs_5x5)
    fprintf('BRL ratios, location %d of %d\n', a, length(Land_coord))
        for c= 1:size(SMARTS_irr_clear_sky_area{a}.Direct_tilted_irradiance,2)
            if ~isnan(input_dr{a,1}(c,1))
            % Share of direct component on TILTED surface:
            input_dr_tilted{a,1}(c,1) = ((input_dr{a,1}(c,1)...
            .*sind(abs(Land_coord(a,1))+90-SMARTS_zenith{a,1}(c,1))) ./
((input_dr{a,1}(c,1).*sind(abs(Land_coord(a,1))+90-SMARTS_zenith{a,1}(c,1)))...
            + (1 - (input_dr{a,1}(c,1).*sind(90-SMARTS_zenith{a,1}(c,1)))) .*
(0.5*(1+cosd(abs(Land_coord(a,1))))));
            % Direct component on TILTED surface considering BRL share:
            G_direct_1h_2019_all_BRL{a,1}(:,c) = ...
(SMARTS_irr_clear_sky_area{a,1}.Direct_tilted_irradiance(:,c)).*(input_dr_tilted
{a,1}(c,1)) ./ (SMARTS_dr_tilted{a,1}(1,c));
            % Direct component on TILTED surface WITHOUT considering BRL share:
             G_direct_1h_2019_all_SMARTS{a,1}(:,c) = ...
            (SMARTS_irr_clear_sky_area{a,1}.Direct_tilted_irradiance(:,c));
```

```
            % Share of diffuse compoent on TIlTED surface:
            input_df_tilted{a,1}(c,1) = (input_df{a,1}(c,1)...
            .*(0.5*(1+cosd(abs(Land_coord(a,1))))) ./ (((1-
input_df{a,1}(c,1)).*sind(abs(Land_coord(a,1))+90-SMARTS_zenith{a,1}(c,1)) ./
sind(90-SMARTS_zenith{a,1}(c,1)))...
            + (input_df{a,1}(c,1).*(0.5*(1+cosd(abs(Land_coord(a,1))))))));
            % Diffuse component on TILTED surface considering BRL share:
            G_diffuse_1h_2019_all_BRL{a,1}(:,c) = ...
            (SMARTS_irr_clear_sky_area{a,1}.Difuse_tilted_irradiance(:,c)).*
(input_df_tilted{a,1}(c,1)) ./ (SMARTS_df_tilted{a,1}(1,c));
             % Diffuse compoent on TIlTED surface WITHOUT considering BRL share:
             G_diffuse_1h_2019_all_SMARTS{a,1}(:,c) = ...
            (SMARTS_irr_clear_sky_area{a,1}.Difuse_tilted_irradiance(:,c));
         % Global component on HORIZONTAL surface WITHOUT considering BRL share:
            GHI_1h_2019_all_SMARTS{a,1}(:,c) = ...
            (SMARTS_irr_clear_sky_area{a,1}.Global_horizn_irradiance(:,c)); else
            end
        end
        mean_zenith{a,1}=mean(SMARTS_zenith{a,1});
end
clear SMARTS_irr_st_part1
save('input_dr_tilted.mat', 'input_dr_tilted', '-v7.3')
save('input_df_tilted.mat', 'input_df_tilted', '-v7.3')
save('G_direct_1h_2019_all_BRL.mat', 'G_direct_1h_2019_all_BRL', '-v7.3')
save('G_diffuse_1h_2019_all_BRL.mat', 'G_diffuse_1h_2019_all_BRL', '-v7.3')
save('G_direct_1h_2019_all_SMARTS.mat', 'G_direct_1h_2019_all_SMARTS', '-v7.3')
save('G_diffuse_1h_2019_all_SMARTS.mat', 'G_diffuse_1h_2019_all_SMARTS','-v7.3')
save('GHI_1h_2019_all_SMARTS.mat', 'GHI_1h_2019_all_SMARTS', '-v7.3')
save('mean_zenith.mat', 'mean_zenith', '-v7.3')

%% Calcuating total plane of array irradiance (GPoA) from different approaches
G_POA_1h_2019_all_BRL=cell(co_max,1); G_POA_1h_2019_all_SMARTS=cell(co_max,1);
for y= 1:length(G_direct_1h_2019_all_BRL)
        for z= 1:size(G_direct_1h_2019_all_BRL{y,1},2)
            % Global component on TILTED surface considering BRL share:
            G_POA_1h_2019_all_BRL{y,1}(:,z)=
G_direct_1h_2019_all_BRL{y,1}(:,z)+G_diffuse_1h_2019_all_BRL{y,1}(:,z);
            % Global component on TILTED surface WITHOUT considering BRL share:
            G_POA_1h_2019_all_SMARTS{y,1}(:,z)=
G_direct_1h_2019_all_SMARTS{y,1}(:,z)+G_diffuse_1h_2019_all_SMARTS{y,1}(:,z);
        end
end
save('G_POA_1h_2019_all_BRL.mat', 'G_POA_1h_2019_all_BRL', '-v7.3')
save('G_POA_1h_2019_all_SMARTS.mat', 'G_POA_1h_2019_all_SMARTS', '-v7.3')
G_POA_1h_2019_avg_raw_BRL = cell(co_max,1);                % prealocating
G_POA_1h_2019_avg_raw_SMARTS = cell(co_max,1);             % prealocating
GHI_1h_2019_avg_raw_SMARTS = cell(co_max,1);               % prealocating
for co= co_min:co_max                   % co=1:length(G_global_1h_2019_all)
G_POA_1h_2019_avg_raw_BRL{co,1}=mean(G_POA_1h_2019_all_BRL{co,1},2, 'omitnan');
G_POA_1h_2019_avg_raw_SMARTS{co,1}=mean(G_POA_1h_2019_all_SMARTS{co,1},2,
'omitnan');
GHI_1h_2019_avg_raw_SMARTS{co,1}=mean(GHI_1h_2019_all_SMARTS{co,1},2,
'omitnan');                         % These are irradiance on TILTED surface!
end
```

```
save('G_POA_1h_2019_avg_raw_BRL.mat', 'G_POA_1h_2019_avg_raw_BRL', '-v7.3')
save('G_POA_1h_2019_avg_raw_SMARTS.mat', 'G_POA_1h_2019_avg_raw_SMARTS', '-
v7.3')
%% normalising
% This is done to find mismatch between SMARTS final daily irradiance output and
CERES data
addpath 'C:\DirectoryOfTheProjectData'
load('wvlngth.mat')
G_POA_mean_BRL = zeros (co_max,1);                              % prealocating
G_POA_mean_SMARTS = zeros (co_max,1); GHI_mean_SMARTS = zeros (co_max,1);
for co=co_min:co_max
    G_POA_mean_BRL(co,1)=trapz (wvlngth,G_POA_1h_2019_avg_raw_BRL{co,1});
    G_POA_mean_SMARTS(co,1)=trapz (wvlngth,G_POA_1h_2019_avg_raw_SMARTS{co,1});
    GHI_mean_SMARTS(co,1)=trapz (wvlngth,GHI_1h_2019_avg_raw_SMARTS{co,1});
end
addpath 'C:\ DirectoryOfTheProjectData\nc files\CERES_BRL_input'
load('sfc_ini_1h_2019_input.mat','sfc_ini_1h_2019_input')
GHI_mean_CERES = zeros (co_max,1);                              % prealocating
for co=co_min:co_max                                            % number of locations
    CERES_sfc=find(sfc_ini_1h_2019_input(co,:)>0);
    GHI_mean_CERES(co,1)=mean(1.*sfc_ini_1h_2019_input(co,CERES_sfc));
    clear CERES_sfc
end
clear sfc_ini_1h_2019_input
nor_factor=GHI_mean_CERES./GHI_mean_SMARTS;         % normalisation factor
nor_factor(isnan(nor_factor))=0;                    % converts Nans to zeros
GHI_1h_2019_avg_normalized=cell(co_max,1); % defines cell array for normalized
irradiance
for co=co_min:co_max
GHI_1h_2019_avg_normalized{co,1}=GHI_1h_2019_avg_raw_SMARTS{co,1}(:,1).*nor_fact
or(co,1);
end
fprintf('Last saving...\n')
save('G_POA_mean_BRL.mat', 'G_POA_mean_BRL', '-v7.3')
save('G_POA_mean_SMARTS.mat', 'G_POA_mean_SMARTS', '-v7.3')
save('GHI_mean_SMARTS.mat', 'GHI_mean_SMARTS', '-v7.3')
save('GHI_mean_CERES.mat', 'GHI_mean_CERES', '-v7.3')
save('nor_factor.mat', 'nor_factor', '-v7.3')
save('GHI_1h_2019_avg_raw_SMARTS.mat', 'GHI_1h_2019_avg_raw_SMARTS', '-v7.3')
save('GHI_1h_2019_avg_normalized.mat', 'GHI_1h_2019_avg_normalized', '-v7.3')
end
```

Methods S8: MATLAB function for incomplete ionization calculations in a crystalline silicon solar cell, related to Step 12.

```
function [Nd_plus, Na_minus, iid, iia] = Incompelte_Ionization (Nd,Na,k,T,n,p)
%% Ionization is calculated based on P. Altermatt et al., J Applied Physics,
2006. (part II of the paper).
q= 1.60217662e-19;                              % Electron charge (Coulombs)
E_dop0_SiP = 45.5; E_dop0_SiB = 44.39;          % meV
Nref_SiP = 3e18; Nref_SiB = 1.7e18;             % cm-3
c_SiP = 2;  c_SiB = 1.4;                        % no unit
Nb_SiP = 6e18; Nb_SiB = 4.5e18;                 % cm-3
d_SiP = 2.3;  d_SiB = 2.4;                      % no unit
g_SiP = 0.5;  g_SiB = 0.25;                     % no unit

b_SiP = 1/(1+(Nd/Nb_SiP)^d_SiP); b_SiB = 1/(1+(Na/Nb_SiB)^d_SiB);
E_dop_SiP = (E_dop0_SiP*1e-3*q)/(1+(Nd/Nref_SiP)^c_SiP);
E_dop_SiB = (E_dop0_SiB*1e-3*q)/(1+(Na/Nref_SiB)^c_SiB);

Nc_300K = 2.89e19;   Nv_300K = 3.14e19;
Nc = Nc_300K * (T/300)^1.5;      Nv = Nv_300K * (T/300)^1.5;  % for crystalline
silicon at 300K, unit is cm-3 obtained from PVlighthouse

n1 = Nc*exp(-E_dop_SiP/(k*T)); p1 = Nv*exp(-E_dop_SiB/(k*T));

Nd_plus = Nd * (1- (b_SiP*n/(n + g_SiP*n1)));
Na_minus= Na * (1- (b_SiB*p/(p + g_SiB*p1)));
iid = Nd_plus/Nd;  iia = Na_minus/Na;
end
```

Methods S9: MATLAB function for calculating statistics of charge carriers in a crystalline silicon solar cell, related to Step 12.

```
function [n0, p0, n, p, delta_n, ni_eff, delta_Eg, Nd_plus, Na_minus, iid, iia]
= Carrier_Statistics(Nd, Na, T, V, ni_0, Eg)
% intrinsic carrier concentration at 25 C is calculated based on A. Richter et
al. IEEE JPV, 2013.

k = 1.38064852e-23;       % Boltzmann constant (m2 kg/s2 K)
q= 1.60217662e-19;        % Electron charge (Coulombs)
Nc_300K = 2.89e19;        % for crystalline silicon at 300K, unit is cm-3
obtained from PVlighthouse
Nv_300K = 3.14e19;       % temperature dependency of Nv and Nc from equations
(14) and (15) of Martin Green. J Applied Physics 67, no. 6 (1990): 2944-2954.
Nc = Nc_300K * (T/300)^1.5;  Nv = Nv_300K * (T/300)^1.5;
N = Nd-Na; n0 = N; n = n0; p = (ni_0^2)/n; t = [0:1e-2:1e2]';
[Nd_plus, Na_minus, iid, iia] = Incompelte_Ionization(Nd, Na, k, T, n, p);
n0 = Nd_plus-Na_minus; n = n0; p = (ni_0^2)/n;
[delta_Eg, delta_Ev, delta_Ec] = Band_Gap_Narrowing(T, Nd_plus, Na_minus, n, p);
x0 = log ((n./Nc)/(1+(n./(4*Nc))));
x1 = log ((n./Nc)/(1+(n./(4*Nc)))) - ((q*delta_Ec)./(k*T));
F_1_2_x0 = (2/sqrt(pi)) * trapz(t, (t*ones(1,length(V)).^0.5) ./ (1+
exp(t*ones(1,length(V))-ones(length(t),1)*x0)));
F_1_2_x1 = (2/sqrt(pi)) * trapz(t, (t*ones(1,length(V)).^0.5) ./ (1+
exp(t*ones(1,length(V))-ones(length(t),1)*x1)));
gamma_deg = F_1_2_x1 / exp(x1);
gamma_BGN = exp (q*delta_Ev./(k*T)) .* (F_1_2_x0 ./ F_1_2_x1);
n0p0 = (ni_0^2) .* gamma_deg .* gamma_BGN;
p0 = n0p0 ./ n0;

%% ni_eff= sqrt(n0p0);
ni_eff = ni_0 * exp ((q*delta_Eg)./(2*k*T));
delta_n = -0.5*(n0 + p0) + 0.5*sqrt(((n0 - p0).^2) +
4*(ni_eff.^2).*exp((q*V)./(k*T)));   % excess carrier concentration should be in
the range of E+11 to E+19 cm-3
delta_n(delta_n<0) = 0;  n = n0 + delta_n;  p = p0 + delta_n;
end
```

Methods S10: MATLAB function to calculate the mobility of electrons and holes in crystalline silicon, related to Step 12.

```
function [mu_e,mu_h,sigma_e,sigma_h,sigma,rho] = Carrier_Mobility (Nd,Na,n,p,T)
% This code calculate carrier mobility, conductance and resistivity
% of silicon based on Arora e. al model (1982)
q= 1.60217662e-19;                       % Electron charge (Coulombs)
N = Nd - Na; Tn = T/300;
mu_e = 88*(Tn^-0.57) + 7.4e8*(T^-2.33) ./ (1+0.88*(Tn^-
0.146).*(N./(1.26e17*(Tn^2.4))));       % electron mobility in silicon (cm2/V.s)
mu_h = 54.3*(Tn^-0.57) + 1.36e8*(T^-2.23) ./ (1+0.88*(Tn^-
0.146).*(N./(2.35e17*(Tn^2.4))));       % hole mobility in silicon (cm2/V.s)
sigma_e = q.*n.*mu_e;                    % conductivity for electrons
sigma_h = q.*p.*mu_h;                    % conductivity for holes
sigma = sigma_e + sigma_h;               % total conductivity
rho = 1./sigma;                          % resistivity  (ohm.cm)
end
```

Methods S11: MATLAB function to calculate the temperature-dependent optical constants of intrinsic silicon, related to Step 12.

```
function [alpha_bb, n_r] = Silicon_Optical_Constants(T)
% temperature dependent of silicon optical coefficients based on M. Green,
SOLMAT, (2008).
load ('Silicon_Absorption_Coeff_300K.mat');         % in supplementary material
T0 = 300;
b = Ck_alpha.*1e-4*T0;                          % b = Cp(T0)T0
alpha_bb = absorption.*((T/T0).^b);      % P(T) = P(T0)*((T/T0)^b)
bb = Cn.*1e-4*T0;
n_r = n_refractive.*((T/T0).^bb);
end
```

Methods S12: MATLAB function to calculate the free carrier absorption in crystalline silicon, related to Step 12.

```
function [alpha_FCA_n,alpha_FCA_p] = Free_Carrier_Absorption(Nd, Na, T, V, n, p)
% Parametrization for free carrier absorption based on Green formulation
% the formulation is described in M. Rudiger el al. IEEE Trans Electron device
(2013). % it is assumed that the doping concentration across the device is
uniform (A. Richter et al. 2013 did the same assumption)
load ('Silicon_Absorption_Coeff_300K.mat');         % in supplementary dataset
k = 1.38064852e-23;           % Boltzmann constant (m2 kg/s2 K)
q= 1.60217662e-19;            % Electron charge (Coulombs)
ni_0 = 8.28e9;                % intrinsic carrier concentration at 25C from the
Richter IEEE JPV paper, 2013.

zeta = 2.6;  phi = 2.4;
C_FCA_n = 1.8e-18; C_FCA_p = 2.6e-18;
alpha_FCA_n = C_FCA_n * ((lamda_um*1e-6./1e-6).^zeta) * n;       % in cm-1
alpha_FCA_p = C_FCA_p * ((lamda_um*1e-6./1e-6).^phi) * p;        % in cm-1
alpha_FCA = alpha_FCA_p + alpha_FCA_n;
end
```

Methods S13: MATLAB function to calculate intrinsic recombination rate in crystalline silicon solar cells, related to Step 12.

```
function [R_intr, Photon_Rec, B_integral] = Intrinsic_Recombination_Rate(Nd, Na,
T, V, W, n, p, n0, p0, delta_n, ni_eff)
% Calculating intrinsic recombination rate (R_intr)
% Coefficients are obtained from P. Altermatt et al. 5th Conference on Numerical
Simulation of Optoelectronic Devices(2005). R_intr equation is obtained from A.
Richter et al. Physical review (2012)
k = 1.38064852e-23;           % Boltzmann constant (m2 kg/s2 K)
q= 1.60217662e-19;            % Electron charge (Coulombs)
ni_0 = 8.28e9;                % intrinsic carrier concentration at 25C from the
Richter IEEE JPV paper, 2013.
%%
b_max = 1.00;       r_max = 0.20;       s_max = 1.5e18;     w_max = 4.0e18;
r_min = 0.00;       s_min = 1.0e7;      w_min = 1.0e9;
b2 = 0.54;   r1 = 320;    s1 = 550;    w1 = 365;
b4 = 1.25;   r2 = 2.50;   s2 = 3.00;   w2 = 3.54;

b_min = r_max + ((r_min - r_max)/(1+((T/r1)^r2)));
b1 = s_max + ((s_min - s_max)/(1+((T/s1)^s2)));
b3 = w_max + ((w_min - w_max)/(1+((T/w1)^w2)));

B_rel = b_min + ((b_max - b_min)./(1+(((n+p)./b1).^b2)+(((n+p)./b3).^b4)));
% from P. Altermatt et al.  5th Conference on Numerical Simulation of
Optoelectronic Devices,(2005)
[Photon_Rec, P_PR, P_FCA, P_ex, B_integral, B_low_integral] =
Photon_Recycling(Nd, Na, T, V, W, n, p, ni_eff);
B_low = B_low_integral;         % radiative recombination coefficient at 300K
% from T. Trupke et al. J applied physics (2003)
B = B_low .* B_rel;             % radiative recombination coefficient at T
N0_eeh = 3.3e17;  %cm^-3        % from A. Richter et al. Physical review (2012)
N0_ehh = 7.0e17;  %cm^-3
g_eeh = 1 + 13*(1-tanh((n0/N0_eeh).^0.66));
g_ehh = 1 + 7.5*(1-tanh((p0/N0_ehh).^0.63));
R_intr = (n.*p - (ni_eff.^2)).*((2.5E-31*g_eeh.*n0) + (8.5E-32*g_ehh.*p0) + (3E-
29.*(delta_n.^0.92)) + (B_rel.*B_low).*(1-Photon_Rec));   % from A. Richter et
al. Physical review (2012)
end
```

Methods S14: MATLAB function to calculate the photon recycling in crystalline silicon solar cells, related to Step 12.

```
function [Photon_Rec, P_PR, P_FCA, P_ex, B_integral, B_low_integral] = 
Photon_Recycling(Nd, Na, T, V, W, n, p, ni_eff)
load ('Silicon_Absorption_Coeff_300K.mat');          % in supplementary dataset
c = 299792458;                      % Speed of light (m/s)
k = 1.38064852e-23;                 % Boltzmann constant (m2 kg/s2 K)
q= 1.60217662e-19;                  % Electron charge (Coulombs)
h = 6.62607004e-34;                % Plack constant (m2 kg / s  or Joule.Second)
ni_0 = 8.28e9;                     % intrinsic carrier concentration at 25C from 
the Richter IEEE JPV paper, 2013.
lamda =1e-6*lamda_um; h_bar = h / (2*pi); E = (h*c)./lamda;
[alpha_bb, n_r] = Silicon_Optical_Constants(T);
[alpha_FCA_n,alpha_FCA_p] = Free_Carrier_Absorption(Nd, Na, T, V, n, p);
alpha_FCA = alpha_FCA_p + alpha_FCA_n;
B_E = ((n_r.^2).*(E.^2).*alpha_bb.*exp(-E./(k*T)))*(1 ./
((pi^2)*((c*100)^2)*(h_bar^3)*(ni_eff.^2)));      % spectrally resolved 
radiative recombination coefficient
A_bb = (alpha_bb*ones(1,length(V))) ./ (alpha_bb*ones(1,length(V)) + alpha_FCA +
(1./(4.*((n_r.^2)*W*1e+2)))*ones(1,length(W)));
P_PR = trapz(flip(E), flip(A_bb.*B_E))./trapz(flip(E), flip(B_E));
B_integral = trapz(flip(E), flip(B_E));
B_lowly_doped = ((n_r.^2).*(E.^2).*alpha_bb.*exp(-E./(k*T)))*(1 ./
((pi^2)*((c*100)^2)*(h_bar^3)*(ni_0.^2)));
B_low_integral = trapz(flip(E), flip(B_lowly_doped));
A_FCA = alpha_FCA ./ (alpha_bb*ones(1,length(V)) + alpha_FCA +
(1./(4.*((n_r.^2)*W*1e+2)))*ones(1,length(W)));
A_ex = ((1./(4.*((n_r.^2)*W*1e+2)))*ones(1,length(W))) ./
(alpha_bb*ones(1,length(V)) + alpha_FCA +
(1./(4.*((n_r.^2)*W*1e+2)))*ones(1,length(W)));
P_FCA = trapz(flip(E), flip(A_FCA.*B_E))./trapz(flip(E), flip(B_E));
P_ex = trapz(flip(E), flip(A_ex.*B_E))./trapz(flip(E), flip(B_E));
Photon_Rec = P_PR;
end
```

Methods S15: MATLAB function to calculate the bandgap narrowing in silicon, related to Step 12.

```
function [delta_Eg, delta_Ev, delta_Ec] = Band_Gap_Narrowing(T, Nd, Na, n, p)
% Bang gap narrowing (BNG) model_Schenk, applied physics 1998.
% Appendix A of K. R. Mcintosh & P. P. Altermatt conference paper 2010
k = 1.38064852e-23; % Boltzmann constant (m2 kg/s2 K)
q= 1.60217662e-19;  % Electron charge (Coulombs)
% Silicon parameter  (Table I of Schenk's paper)
Ry_ex = 16.55e-3;    % in eV unit "exciton Rydberg energy" for silicon
alpha_e = 0.5187;    % mu* to me >>> mu* is reduced effective mass, me is
effective mass of electron
alpha_h = 0.4813;    % mu* to mh >>> mu* is reduced effective mass, mh is
effective mass of hole
g_e = 12;            % degeneracy factor for electron
g_h = 4;             % degeneracy factor for hole
me_m0 = 0.321;       % effective mass of electron to m0 (m0 is the "free electron
rest mass" equals 9.11e-31 kg)
mh_m0 = 0.346;       % effective mass of hole to m0
mu_m0 = 0.1665;      % reduced effective mass to m0
a_ex = 37.19e-8;     % excitonic Bohr radius in cm
epsilon_s = 11.7;    % static dielectric constant
% electronic (rigid) part   (Table II of Schenk's paper)
b_e = 8;             % Fit parameters for Pade approximation for electronic BGN
equation, for both electrons and holes
b_h = 1;     c_e = 1.3346;       c_h = 1.2365;       d_e = 0.893;
d_h = 1.153; p_e = 7/30;         p_h = 7/30;
% ionic part        (Table III of Schenk's paper)
% Fit parameters of Pade approximation for ionic BGN equation, for both
electrons and holes
h_e = 3.91;         h_h = 4.20;         j_e = 2.8585;       j_h = 2.9307;
k_e = 0.012;        k_h = 0.19;         q_e = 0.75;         q_h = 0.25;
%% CALCULATIONS
% Calculations of carrier-related parameters
n_e = (a_ex^3)*n; n_h = (a_ex^3)*p; n_p = (alpha_e*n_e) + (alpha_h*n_h);
n_sigma = n_e + n_h; zeta = (k*T)/(Ry_ex*q);  U_i = (n_sigma.^2)./(zeta^3);
N_sigma = Nd + Na; n_ionic = (a_ex^3)*N_sigma;   % impurity concentration

% calculation of band shifts
delta_e_i = (-n_ionic.*(1+U_i)) ./
(sqrt((zeta.*n_sigma)./(2*pi)).*(1+h_e.*log(1+(sqrt(n_sigma)./zeta))) +
j_e.*U_i.*((n_p).^0.75).*(1+k_e.*(n_p.^q_e)));
delta_h_i = (-n_ionic.*(1+U_i)) ./
(sqrt((zeta.*n_sigma)./(2*pi)).*(1+h_h.*log(1+(sqrt(n_sigma)./zeta))) +
j_h.*U_i.*((n_p).^0.75).*(1+k_h.*(n_p.^q_h)));
delta_Ec_i = -Ry_ex.*delta_e_i; %ionic quasi-particle shift of conduction band
delta_Ev_i = -Ry_ex.*delta_h_i; %ionic quasi-particle shift of valence band

delta_e_xc = -(((4*pi)^3)*((n_sigma.^2).*(((48*n_e)./(pi*g_e)).^(1/3)+
c_e*log(1+d_e.*(n_p.^p_e)))) + (8*pi*alpha_e/g_e).*n_e.*(zeta^2) +
sqrt(8*pi.*n_sigma).*(zeta^2.5)) / (((4*pi)^3)*(n_sigma.^2) + zeta^3 +
b_e*sqrt(n_sigma).*(zeta^2) + 40*(n_sigma.^1.5).*zeta);
```

```
delta_h_xc = -(((4*pi)^3)*((n_sigma.^2).*(((48*n_h)./(pi*g_h)).^(1/3)+
c_h*log(1+d_h.*(n_p.^p_h)))) + (8*pi*alpha_h/g_h).*n_h.*(zeta^2) +
sqrt(8*pi.*n_sigma).*(zeta^2.5)) / (((4*pi)^3)*(n_sigma.^2) + zeta^3 +
b_h*sqrt(n_sigma).*(zeta^2) + 40*(n_sigma.^1.5).*zeta);

delta_Ec_xc = -Ry_ex.*delta_e_xc; % electronics (rigid) quasi-particle shift of
conduction band
delta_Ev_xc = -Ry_ex.*delta_h_xc; % electronics (rigid) quasi-particle shift of
valence band
delta_Ec = delta_Ec_xc + delta_Ec_i;   delta_Ev = delta_Ev_xc + delta_Ev_i;
BGN = delta_Ec + delta_Ev;             delta_Eg = abs(BGN);
end
```

Methods S16: MATLAB function to calculate the 2 terminal double-junction silicon-based tandem solar cell parameters and efficiency under real world conditions, related to Step 13.

```
function [ eta_tandem ] = tandem_2T_world_PSO(bandgap, Width, N_donor,
LandCoordIndex,...
lamda_nm,Tair_C_Daily_avg_2019_input,G_POA_mean_BRL_world,Coef_WeightAveToAve_GP
oA,Coef_WeightAveToAve_Tair,G_POA_1h_2019_avg_raw_BRL_world)
% This function calculate solar cell theoretical efficiency of X-Si tandem 2-
termainal solar cell % The top cell is calculated using detailed balanced limit
and the bottom cell using 1-D semiconductor modelling
c = 299792458;                   % Speed of light (m/s)
k = 1.38064852e-23;              % Boltzmann constant (m2 kg/s2 e1)
q= 1.60217662e-19;               % Electron charge (Coulombs)
h = 6.62607004e-34;              % Plack constant (m2 kg / s  or Joule.Second)
load ('WorldData_OneTime_Load.mat');                 % in supplementary dataset
N_acceptor = 0; NOCT = 48; Vstart=0.0; Vstep=0.0002; lamda =1e-9*lamda_nm;
Result = zeros(1,27); % prealocation
E_global_lamda = G_POA_1h_2019_avg_raw_BRL_world{LandCoordIndex,1}; % irradiance
Tc = 273.15 + Tair_C_Daily_avg_2019_input(LandCoordIndex,1) +...
    (((NOCT-20)/800))*G_POA_mean_BRL_world(LandCoordIndex,1); % temp. Kelvin
phi_lamda = ((q.*lamda)./(h*c)).*(E_global_lamda); % converts spectral
irradiance energy into spectral photon flux (C/m2 s)
total_incoming_radiation_top = trapz(lamda_nm, E_global_lamda);     % incoming
radiation on the top cell

%% Simulating the top cell based on detailed balance limit approach
Fg = 2;      % geometrical factor (Solar cell emits radiation from both sides).
counter = 1:1:length(bandgap); Eg = q * bandgap(counter,1); lamda_g = (h*c)./Eg;
unitstep = zeros(length(lamda),1);  absorptance = zeros(length(lamda),1);
for j=1:length(Eg)               % to make a step function for absorptance
unitstep (:,j) = lamda>lamda_g(j,1);
absorptance (:,j) = ones(length(lamda),1)-unitstep(:,j);
end
J_max_Eg = trapz (lamda_nm, absorptance.*phi_lamda);
voltage = [Vstart:Vstep:bandgap(counter,1)]';
Jr_Eg_V = zeros(length(voltage),1);          % prealocation
J_ext_Eg_V = zeros(length(voltage),1);       % prealocation
Power_ext_Eg_V = zeros(length(voltage),1);   % prealocation
for j=1:length(Eg)
    Vend=Eg(j,1)/q;  i=1;
for V=Vstart:Vstep:Vend
Jr_Eg_V (i,j) = Fg * (-q) * trapz (E, (2*pi.*(E.^2)./((h^3)*(c^2))) .*
(absorptance(:,j)./(exp((E-(q*V))./(k*Tc))-1)));    % to find radiative
recombination
J_ext_Eg_V (i,j) = J_max_Eg (1,j) - Jr_Eg_V (i,j);
Power_ext_Eg_V (i,j) = (V.*J_ext_Eg_V (i,j)); i=i+1;
end
if j/length(Eg)>0.5 && j/length(Eg)<0.51  % to monitor the loop progress
fprintf('first loop, halfway through, progress: %.1f \n %', (j/length(Eg))*100)
    end
end
J_ext_Eg_V(J_ext_Eg_V<=0) = nan;
minimum = zeros(1,length(Eg)); maximum = zeros(1,length(Eg));     % prealocation
x = zeros(1,length(Eg));  y = zeros(1,length(Eg));                % prealocation
Voc = zeros(1,length(Eg)); Vmpp = zeros(1,length(Eg));            % prealocation
```

```
for j=1:length(Eg)
minimum (1,j) = min(J_ext_Eg_V(:,j));                             % to find Voc
x(1,j)=find(J_ext_Eg_V(:,j)==minimum(1,j)); Voc(1,j)=Vstart+(Vstep.*(x(1,j)-1));
maximum (1,j) = max(Power_ext_Eg_V(:,j));                         % to find Vmpp
y(1,j)=find(Power_ext_Eg_V(:,j)==maximum(1,j));
Vmpp (1,j) = Vstart+(Vstep.*(y(1,j)-1));
end
Jr_Eg_Vmpp = zeros(1,length(Eg)); J_mpp_Eg = zeros(1,length(Eg)); % prealocation
Jr_Eg_0 = zeros(1,length(Eg));  J_sc_Eg = zeros(1,length(Eg));    % prealocation
for j=1:length(Eg)
Jr_Eg_Vmpp (1,j) = Fg * (-q) .* trapz (E, (2*pi.*(E.^2)./((h^3)*(c^2))) .*
(absorptance(:,j)./(exp((E-(q.*Vmpp(1,j)))./(k*Tc))-1)));
J_mpp_Eg (1,j) = J_max_Eg (1,j) - Jr_Eg_Vmpp (1,j);
Jr_Eg_0 (1,j) = Fg * (-q) .* trapz (E, (2*pi.*(E.^2)./((h^3)*(c^2))) .*
(absorptance(:,j)./(exp((E-(q*0))./(k*Tc))-1)));
J_sc_Eg (1,j) = J_max_Eg (1,j) - Jr_Eg_0 (1,j);
end
Voc_top = Voc; Jsc_top = J_sc_Eg; Vmpp_top = Vmpp; Jmpp_top = J_mpp_Eg;
FF_top = (Vmpp.*J_mpp_Eg)./(Voc.*J_sc_Eg);
eta_top = (Vmpp.*J_mpp_Eg)./total_incoming_radiation_top;
%% Simulating the bottom cell based on semi-conductor physics equations of
silicon. This is similar to the single junction silicon code
V = [Vstart:Vstep:bandgap(counter,1)]; T=Tc;
ni_0 = 5.29e19 * ((T/300)^2.54) * exp(-6726/T) ;
phi_lamda_bottom = (1-absorptance).*((q.*lamda)./(h*c)).*(E_global_lamda);
total_incoming_radiation_bottom=trapz(lamda_nm,(1-absorptance).*E_global_lamda);
% incoming radiation for the bottom cell
%% Pre-allocation
Pmpp = zeros (length(N_donor), length(Width));
eta_bottom = zeros (length(N_donor), length(Width));
Jsc_mApercm2 = zeros (length(N_donor), length(Width));
Voc = zeros (length(N_donor), length(Width));
FF = zeros (length(N_donor), length(Width));
delta_n_mpp = zeros (length(N_donor), length(Width));
Tau_mpp = zeros (length(N_donor), length(Width));
Tau_oc = zeros (length(N_donor), length(Width));
delta_n_oc = zeros (length(N_donor), length(Width));
Vmpp = zeros (length(N_donor), length(Width));
Resistivity_sc = zeros (length(N_donor), length(Width));
Resistivity_oc = zeros (length(N_donor), length(Width));
Resistivity_mpp = zeros (length(N_donor), length(Width));
L_mpp = zeros (length(N_donor), length(Width));
L_oc = zeros (length(N_donor), length(Width));
Photon_Rec_Width = zeros (length(Width), length(V));
B_integral_Width = zeros (length(Width), length(V));
BGN_values = zeros (length(N_donor), length(V));
ni_eff_values = zeros (length(N_donor), length(V));
delta_n_values = zeros (length(N_donor), length(V));
x = zeros (1, length(Width)); y = zeros (1, length(Width));
Nd_plus = zeros (length(N_donor), 1); Na_minus = zeros (length(N_donor), 1);
```

```
minimum = zeros (1, length(Width));
eta_tandem = zeros (length(N_donor), length(Width));
Voc_tandem = zeros (length(N_donor), length(Width));
Jsc_tandem = zeros (length(N_donor), length(Width));
Vmpp_tandem = zeros (length(N_donor), length(Width));
Jmpp_tandem = zeros (length(N_donor), length(Width));
radiation_top = zeros (length(N_donor), length(Width));
radiation_bottom = zeros (length(N_donor), length(Width));
NoOutputBottomCell_loop_abort_counter=0; width_loop_abort_counter=0;
%% Loop
for j = 1:1:length(N_donor)
Nd = N_donor(1,j); Na = N_acceptor;
% Incomplete Ionization and carrier concentration functions are skipped in the
tandem calculations as they make minor influence on the overall final results.
% [Nd_plus, Na_minus] = Incompelte_Ioization(Nd, Na, k, T, n, p);
% [n0, p0, n, p, delta_n, ni_eff, delta_Eg] = Carrier_Concentration(Nd, Na, T,
V, ni_0, Eg);
[n0, p0, n, p, delta_n, ni_eff, delta_Eg, Nd_plus(j,1), Na_minus(j,1), ~, ~] =
Carrier_Statistics(Nd, Na, T, V, ni_0, Eg);
BGN_values (j,:) = delta_Eg; ni_eff_values (j,:) = ni_eff;
delta_n_values (j,:) = delta_n;  ni_eff (:,1);  delta_Eg (:,1);
[~, mu_h, ~, ~, ~, rho] = Carrier_Mobility (Nd, Na, n, p, T);
[alpha_bb, n_r] = Silicon_Optical_Constants(T);
[alpha_FCA_n,alpha_FCA_p] = Free_Carrier_Absorption(Nd, Na, T, V, n, p);
alpha_FCA = alpha_FCA_p + alpha_FCA_n;

for i = 1:1:length(Width)
W = Width(1,i);
[R_intr, Photon_Rec, B_integral] = Intrinsic_Recombination_Rate(Nd, Na, T, V, W,
n, p, n0, p0, delta_n, ni_eff);
Photon_Rec_Width(i,:) = Photon_Rec; B_integral_Width(i,:) = B_integral;
A_bb = (alpha_bb*ones(1,length(V))) ./ (alpha_bb*ones(1,length(V)) + alpha_FCA +
(1./(4.*((n_r.^2)*W*1e+2)))*ones(1,length(W)));
J_L_mApercm2 = (1e3/1e4).*trapz(lamda_nm,
A_bb.*(phi_lamda_bottom*ones(1,length(V))));
J_mApercm2 = (J_L_mApercm2) - 1000*q*((W*1e+2).*real(R_intr));
J_Aperm2 = J_mApercm2*10; P = J_Aperm2.*V;  Pmpp(j,i) = max(P);
eta_bottom(j,i) = (Pmpp(j,i))./total_incoming_radiation_bottom;

Jsc_mApercm2 (j,i) = max (J_mApercm2); minimum (1,i) = min(abs(J_mApercm2));
x(1,i)=find(abs(J_mApercm2) == minimum(1,i),1, 'last');    % this finds the last
value that matches the condition
Voc (j,i) = Vstart+(Vstep.*(x(1,i)-1));
FF (j,i) = Pmpp(j,i) / (10.*(Voc(j,i)*Jsc_mApercm2(j,i)));
y(1,i)=find(P == Pmpp(j,i));
delta_n_mpp(j,i) = delta_n(1, y(1,i));
Tau_mpp(j,i) = delta_n_mpp(j,i)/R_intr(1, y(1,i));
delta_n_oc(j,i) = delta_n(1, x(1,i));
Tau_oc(j,i) = delta_n_oc(j,i)/R_intr(1, x(1,i)); Vmpp(j,i) = V(1, y(1,i));
Resistivity_sc (j,i)= rho(1,1); Resistivity_oc (j,i)= rho(1,x(1,i));
Resistivity_mpp (j,i)= rho(1,y(1,i));
L_mpp(j,i) = sqrt((k*T/q)*(mu_h*Tau_mpp(j,i)));
L_oc(j,i) = sqrt((k*T/q)*(mu_h*Tau_oc(j,i)));
```

```
%% finding the JV curve of the 2 terminal tandem cell (series connection)
JV_top = [voltage 0.1*J_ext_Eg_V];      % converts A/m2 to mA/cm2
JV_bottom = [V' 0.1*J_Aperm2'];         % converts A/m2 to mA/cm2
JV_top(isnan(JV_top(:,2)),:)=[];        % removes nans from JV top
JV_bottom(JV_bottom(:,2)<0,:)=[];       % removes negative values from JV bottom

if isempty(JV_bottom)  % if bottom cell gives no output (all light absorbed in
the top cell), record all tandem cell values as zero and abort the loop
Voc_tandem(j,i) = 0; Jsc_tandem(j,i) = 0; Vmpp_tandem(j,i) = 0;
Jmpp_tandem(j,i) = 0; radiation_top(j,i) = total_incoming_radiation_top;
radiation_bottom(j,i) = total_incoming_radiation_bottom;
NoOutputBottomCell_loop_abort_counter = NoOutputBottomCell_loop_abort_counter+1;
        continue   % skips the rest of the calculation and goes to the next
iteration of this loop
end

if max(JV_bottom(:,2)) >= max(JV_top(:,2))   % The JV with the smaller Isc must
be extended (as extended as the Voc of the other curve) and the other JV must be
flipped. In this way the intersect will happen in any case.
J_extrapolate = interp1(JV_top(:,1), JV_top(:,2), [-
max(JV_bottom(:,1)):Vstep:max(JV_top(:,1))]','linear','extrap');
JV_extrapolate = [[-max(JV_bottom(:,1)):Vstep:max(JV_top(:,1))]' J_extrapolate];
    JV_flipped = [-JV_bottom(:,1) JV_bottom(:,2)];
else
J_extrapolate = interp1(JV_bottom(:,1), JV_bottom(:,2), [-
max(JV_top(:,1)):Vstep:max(JV_bottom(:,1))]','linear','extrap');
JV_extrapolate = [[-max(JV_top(:,1)):Vstep:max(JV_bottom(:,1))]' J_extrapolate];
    JV_flipped = [-JV_top(:,1) JV_top(:,2)];
end

decimal_points = 3;
for decimal_counter = 1:decimal_points+1
[C, ~, ib] =
intersect(round(JV_extrapolate(:,2),decimal_points),round(JV_flipped(:,2),decima
l_points));         % to find the intersection point
if ~isempty(C)      % we reduce the finding intersection accuracy step by step
from 3 decimal points to zero decimal point
break
else
   decimal_points=decimal_points-1;
end
end
if isempty(C)       % if there is still no intersection for some reason, just
abort this width loop and go to the next thickness width
        width_loop_abort_counter = width_loop_abort_counter+1;
        break
end

% now we just need to shift the curve
voltage_shift_size = abs(JV_flipped(ib(end),1));
JV_tandem = [JV_extrapolate(:,1)+voltage_shift_size JV_extrapolate(:,2)];
JV_tandem(JV_tandem(:,1)<0,:)=[];  % removes negative values of voltage
```

```
PV_tandem = [JV_tandem(:,1) 10*(JV_tandem(:,1).*JV_tandem(:,2))];     % converts
back from mA/cm2 to A/m2
[row, ~] = find(PV_tandem(:,2)==max(PV_tandem(:,2)));
eta_tandem (j,i) = PV_tandem(row,2)/total_incoming_radiation_top;
Voc_tandem(j,i) = JV_tandem(end,1); Jsc_tandem(j,i) = JV_tandem(1,2);
Vmpp_tandem(j,i) = JV_tandem(row,1); Jmpp_tandem(j,i) = JV_tandem(row,2);
radiation_top(j,i) = total_incoming_radiation_top;
radiation_bottom(j,i) = total_incoming_radiation_bottom;
end
end
%% Saving
[index_row,index_column]=find(eta_tandem==max(max(eta_tandem)),1,'first');
Result(counter,:) = [eta_bottom(index_row,index_column)
Voc(index_row,index_column) Jsc_mApercm2(index_row,index_column)
100*FF(index_row,index_column)...
        Vmpp(index_row,index_column) delta_n_mpp(index_row,index_column)
delta_n_oc(index_row,index_column) 1e6*Width(1,index_column)...
        BGN_values(index_row,1) ni_eff_values(index_row,1) Nd_plus(index_row,1)-
Na_minus(index_row,1) Resistivity_mpp(index_row,index_column)...
        (1e4*L_mpp(index_row,index_column))/(1e6*Width(1,index_column))
eta_tandem(index_row,index_column) Voc_tandem(index_row,index_column)...
        Jsc_tandem(index_row,index_column) Vmpp_tandem(index_row,index_column)
Jmpp_tandem(index_row,index_column) radiation_top(index_row,index_column)...
        radiation_bottom(index_row,index_column) bandgap(counter,1) eta_top
Voc_top Jsc_top Vmpp_top Jmpp_top 100*FF_top];
eta_tandem = Result(1,14);
end
```

Methods S17: MATLAB function to calculate solar cell parameters and efficiency using the detailed balance limit approach, related to Step 13.

```
function [ eta ] = Detailed_Limit( lamda_nm, E_global_lamda, Tc, Eg, Fg)
% This function calculate solar cell theoretical efficiency using detailed
balance approach. % Equations and results from this function is cross checked
with the paper: tabulated values of Shockley-Queisser limit for single junction
solar cells (Solar Energy 2016).
clear all;
c = 299792458;                  % Speed of light (m/s)
k = 1.38064852e-23;             % Boltzmann constant (m2 kg/s2 e1)
q= 1.60217662e-19;              % Electron charge (Coulombs)
h = 6.62607004e-34;             % Plack constant (m2kg/s  or Joule.Second)
addpath('C:\DirectoryOfTheProjectData');
load ('ASTMG173.mat');  % 'lamda_nm' , 'E_global_lamda', and 'E_beam_lamda' are
the variable inputs from ASTMG173                    % in supplementary dataset
lamda =1e-9*lamda_nm;
Eg = q * input('insert the bandgap of the material in eV (Eg)  = ');
% could be a number (e.g. 1.12) or a vector such as: Eg=[0.31:0.01:4.42]';
Fg = input('insert the geometrical factor for solar cell emitting radiation:
either 1 or 2 sides (Fg)  = ');
Tc = input('insert the solar cell temperature in Kelvin (Tc)  = ');
n = length(lamda);            % returns number of elements in the Lamda vector
phi_lamda = ((q.*lamda)./(h*c)).*(E_global_lamda);
% converts AM 1.5 spectral irradiance into spectral photon flux (C/m2 s)
lamda_g = (h*c)./Eg; lamda_nm_g = lamda_g .* 1e9;

for j=1:length(Eg)              % to make a step function for absorptance
    unitstep (:,j) = lamda>lamda_g(j,1);
    absorptance (:,j) = ones(n,1)-unitstep (:,j);
end

J_max_Eg = trapz (lamda_nm, absorbtance.*phi_lamda);
J_max_Eg_mApercm2 = (1e3/1e4).*J_max_Eg;      % converts A/m2 to mA/cm2
E = h*c./lamda;                               % energy of each photon
Jnr_Eg_V = 0;  % non-radiative recombination is neglected in detailed balance
Jnr_Eg_0 = 0;
Vstart=0.001; Vstep=0.0005;
for j=1:length(Eg)
    Vend=Eg(j,1)/q; i=1;
for V=Vstart:Vstep:Vend
Jr_Eg_V (i,j) = Fg * (-q) * trapz (E, (2*pi.*(E.^2)./((h^3)*(c^2))) .*
(absorbtance(:,j)./(exp((E-(q*V))./(k*Tc))-1))); % finds radiative recombination
J_ext_Eg_V (i,j) = J_max_Eg (1,j) - Jr_Eg_V (i,j);
Power_ext_Eg_V (i,j) = (V.*J_ext_Eg_V (i,j)); i=i+1;
end
    if j/length(Eg)>0.5 && j/length(Eg)<0.51
%to monitor the loop progress
    fprintf('first loop, halfway through, progress: %.1f \n %',
(j/length(Eg))*100)
    end
end
disp('first loop, done.')                            % to monitor the loop progress
J_ext_Eg_V(J_ext_Eg_V<=0) = nan;
```

```
for j=1:length(Eg)
minimum (1,j) = min(J_ext_Eg_V(:,j));                          % to find Voc
x(1,j)=find(J_ext_Eg_V(:,j)==minimum(1,j));
Voc (1,j)= Vstart+(Vstep.*(x(1,j)-1));

maximum (1,j) = max(Power_ext_Eg_V(:,j));                     % to find Vmpp
y(1,j)=find(Power_ext_Eg_V(:,j)==maximum(1,j));
Vmpp (1,j) = Vstart+(Vstep.*(y(1,j)-1));
end

for j=1:length(Eg)
Jr_Eg_Vmpp (1,j) = Fg * (-q) .* trapz (E, (2*pi.*(E.^2)./((h^3)*(c^2))) .*
(absorbtance(:,j)./(exp((E-(q.*Vmpp(1,j)))./(k*Tc))-1)));
J_mpp_Eg (1,j) = J_max_Eg (1,j) - Jr_Eg_Vmpp (1,j);

Jr_Eg_0 (1,j) = Fg * (-q) .* trapz (E, (2*pi.*(E.^2)./((h^3)*(c^2))) .*
(absorbtance(:,j)./(exp((E-(q*0))./(k*Tc))-1)));
J_sc_Eg (1,j) = J_max_Eg (1,j) - Jr_Eg_0 (1,j);
end
disp('second loop, done.')                        % to monitor the loop progress

FF = (Vmpp.*J_mpp_Eg)./(Voc.*J_sc_Eg);
%Fill Factor
total_incoming_radiation = trapz(lamda_nm, E_global_lamda);
eta = (Vmpp.*J_mpp_Eg)./total_incoming_radiation;            % SQ efficiency

save ('Detailed_Limit')
end
```
